# Kinetics of PTEN-mediated PI(3,4,5)P3 hydrolysis on solid supported membranes

**DOI:** 10.1371/journal.pone.0192667

**Published:** 2018-02-15

**Authors:** Chun Liu, Sanghamitra Deb, Vinicius S. Ferreira, Eric Xu, Tobias Baumgart

**Affiliations:** Department of Chemistry, School of Arts & Sciences, University of Pennsylvania, Philadelphia, Pennsylvania, United States of America; National Cancer Institute, UNITED STATES

## Abstract

Phosphatidylinositides play important roles in cellular signaling and migration. Phosphatidylinositol-3,4,5-trisphosphate (PI(3,4,5)P3) is an important phosphatidylinositide because it acts as a secondary messenger to trigger cell movement and proliferation. A high level of PI(3,4,5)P3 at the plasma membrane is known to contribute to tumorigenesis. One key enzyme that regulates PI(3,4,5)P3 levels at the plasma membrane is phosphatase and tensin homologue deleted on chromosome 10 (PTEN), which dephosphorylates PI(3,4,5)P3 through hydrolysis to form phosphatidylinositol-4,5-bisphosphate (PI(4,5)P2). It has been reported that PI(4,5)P2 is involved in positive feedback in the PI(3,4,5)P3 hydrolysis by PTEN. However, how PI(3,4,5)P3 dephosphorylation by PTEN is regulated, is still under debate. How other PI(3,4,5)P3-binding proteins affect the dephosphorylation kinetics catalyzed by PTEN also remains unclear. Here, we develop a fluorescent-protein biosensor approach to study how PI(3,4,5)P3 dephosphorylation is regulated by PTEN as well as its membrane-mediated feedback mechanisms. Our observation of sigmoidal kinetics of the PI(3,4,5)P3 hydrolysis reaction supports the notion of autocatalysis in PTEN function. We developed a kinetic model to describe the observed reaction kinetics, which allowed us to i) distinguish between membrane-recruitment and allosteric activation of PTEN by PI(4,5)P2, ii) account for the influence of the biosensor on the observed reaction kinetics, and iii) demonstrate that all of these mechanisms contribute to the kinetics of PTEN-mediated catalysis.

## Introduction

The phosphatase PTEN (phosphatase and tensin homologue deleted on chromosome 10) regulates the well-known PI3K / AKT pathway that is central in many cellular processes including cell growth, differentiation, and apoptosis [1]. PTEN hydrolyses the phosphatidylinositol-3,4,5-trisphosphate PI(3,4,5)P3 at the 3-OH position of the inositol ring. This process down-regulates membrane binding and subsequent activation of the serine-threonine protein kinase AKT (also called protein kinase B), which has many cellular downstream effectors. Up-regulation of this pathway occurs through phosphoinositide 3-OH kinases (PI3K).

PTEN is known as a tumor suppressor that shows a loss of activity through mutations or varied expression levels in many types of cancer. PTEN is involved in several additional diseases, including autism, macrocephaly, and Cowden syndrome [2, 3]. The structure of PTEN is similar to that of dual specificity protein phosphatases, but contains an active site pocket with a larger width compared to the size of phospho-tyrosine, -serine, or -threonine residues, to be able to accommodate the PI(3,4,5)P3 headgroup [4]. In addition to the catalytic domain, PTEN contains a C2 domain that facilitates membrane binding electrostatically through a hopping type interaction [5]. Furthermore, PTEN contains the catalytic signature motif of protein tyrosine phosphatases, which includes a cysteine residue that engages in nucleophilic attack of the substrate to accomplish cleavage [6].

While most of the PTEN contained within cells is localized in the cytoplasm, PTEN’s best characterized enzymatic action occurs at the inner leaflet of the plasma membrane, where the substrate PI(3,4,5)P3 is localized [7]. In addition to the substrate, the membrane binding of PTEN involves (at least) two additional lipid types. The C2 domain of PTEN interacts with negatively charged lipids, such as phosphatidylserine [8]. Furthermore, PTEN possesses an N-terminal phosphatidylinositol-4,5-bisphosphate PI(4,5)P2 binding domain. This third interaction likely gives rise to two different mechanisms by which the catalytic activity of PTEN is modulated through enzymatic product formation. The first mechanism involves an increase in membrane binding affinity through product formation [9]. The second one involves a conformational change of the enzyme after PI(4,5)P2 binding that causes allosteric activation [10, 11]. To what extent these two mechanisms contribute to the overall autocatalytic kinetics of PTEN catalyzed PI(3,4,5)P3 conversion is currently unknown. To clarify this matter is one of the main objectives of this contribution.

What is the importance of autocatalytic hydrolysis of PI(3,4,5)P3 by PTEN? Gamba et al., performed computer simulations to show that the spatial segregation of membrane-associated signaling molecules observed in eukaryotic chemotaxis under either anisotropic or isotropic chemoattractant stimulation is the result of fluctuations in PI(3,4,5)P3 / PI(4,5)P2 concentration and the autocatalysis of PTEN [12]. These results suggested that this autocatalytic regulation is important in cell polarization. Consistent with this notion, Arai et al. showed that PTEN autocatalysis is important in spatiotemporal oscillations of phosphatidylinositol lipid concentrations in the membrane [13].

On the other hand, signaling proteins bearing a pleckstrin homology (PH) domain with PI(3,4,5)P3 binding specificity also play important roles in regulating cell survival and directional movement [14]. The serine-threonine kinase AKT, for example, binds to the membrane via PI(3,4,5)P3, a process which facilitates its activation by phosphoinositide-dependent kinase 1 (PDK 1). The activated AKT phosphorylates downstream proteins, which ultimately stimulates cell growth. The general receptor for 3-phosphoinositide (Grp1) and ADP-ribosylation factor nucleotide binding site opener (ARNO) are two guanine nucleotide exchange factors (GEFs) of ADP-ribosylation factor 6 (ARF6), which is an important mediator of cytoskeleton remodeling [15]. Both Grp1 and ARNO are recruited to the membrane by the interaction of a PH-domain and membrane phosphoinositides, especially PI(3,4,5)P3 [16, 17]. How Grp1 and other proteins bearing PI(3,4,5)P3–specific PH domains affect PI(3,4,5)P3 dephosphorylation is not clear. Characterizing how the rate of PI(3,4,5)P3 dephosphorylation by PTEN is regulated by PH domain-containing proteins is an additional aim of this contribution.

Several previous studies have analyzed the enzyme kinetics of PTEN. Initial studies assessed the hydrolysis of the highly soluble inositol 1,3,4,5-tetrakisphosphate substrate based on a radioactivity assay [18]. The malachite green assay [19] can be used for both soluble substrates [20] and those embedded in lipid membranes [19]. A method based on a soluble fluorescent substrate has also been developed [21]. While the assessment of PTEN activity by means of soluble substrates is at times efficient and straightforward, it has been appreciated for a long time that the activity of interfacial enzymes, including PTEN can be substantially affected by the interface [22, 23]. Accordingly, several previous contributions have studied PTEN catalysis by means of phosphoinositide lipids contained in vesicles [9, 24]. One challenge of working with a dispersion of vesicles containing mixed lipids is compositional variation from vesicle to vesicle [25].

This problem can be solved by working with single lipid bilayer membranes deposited onto a solid support [26]. Solid supported membranes, in either solely physisorbed [7] or partially tethered form [27], have previously been used to study the membrane binding behavior of PTEN. This approach allows the application of surface sensitive techniques, including surface plasmon resonance spectroscopy [27] and total internal reflection microscopy imaging [28]. The application of optical imaging to study PTEN action may ultimately allow the study of spatio-temporal dynamics of reconstituted versions of the PI3K / PTEN reaction pathway [29, 30].

Fluorescence imaging techniques require the use of fluorophores. In the case of the PI3K / AKT reaction pathway, pleckstrin homology (PH) domains are commonly used as biosensors for specific phosphoinositide lipids [13]. However, the presence of the biosensor can, of course, affect reaction kinetics [31]. Therefore, in this contribution we develop an experimental approach that allows us to measure local PH domain concentrations to assess enzyme kinetics, and to account for the presence of the biosensor in determining PI(3,4,5)P3 hydrolysis kinetics. Our kinetic analysis approach surpasses the usual application of Michaelis-Menten type interfacial catalysis models [32] that are based on steady-state assumptions and allows us to determine numerous kinetic parameters of interest to the study of PTEN function.

This contribution is organized as follows. We first describe the design and characterization of a lipid bilayer membrane-containing flow chamber that we use to study PTEN enzyme kinetics on membranes. We then characterize the membrane interaction kinetics of the PI(3,4,5)P3 sensor YFP-PHGrp1 for our experimental conditions. Next we use this sensor to elucidate PTEN catalysis and discuss mechanistic aspects of PTEN function. Our measurements are analyzed with a kinetic model that accounts for the effect of the PI(3,4,5)P3 sensor on PTEN-mediated reaction kinetics.

## Results

### Design and characterization of the experimental system

**Fig 1:**
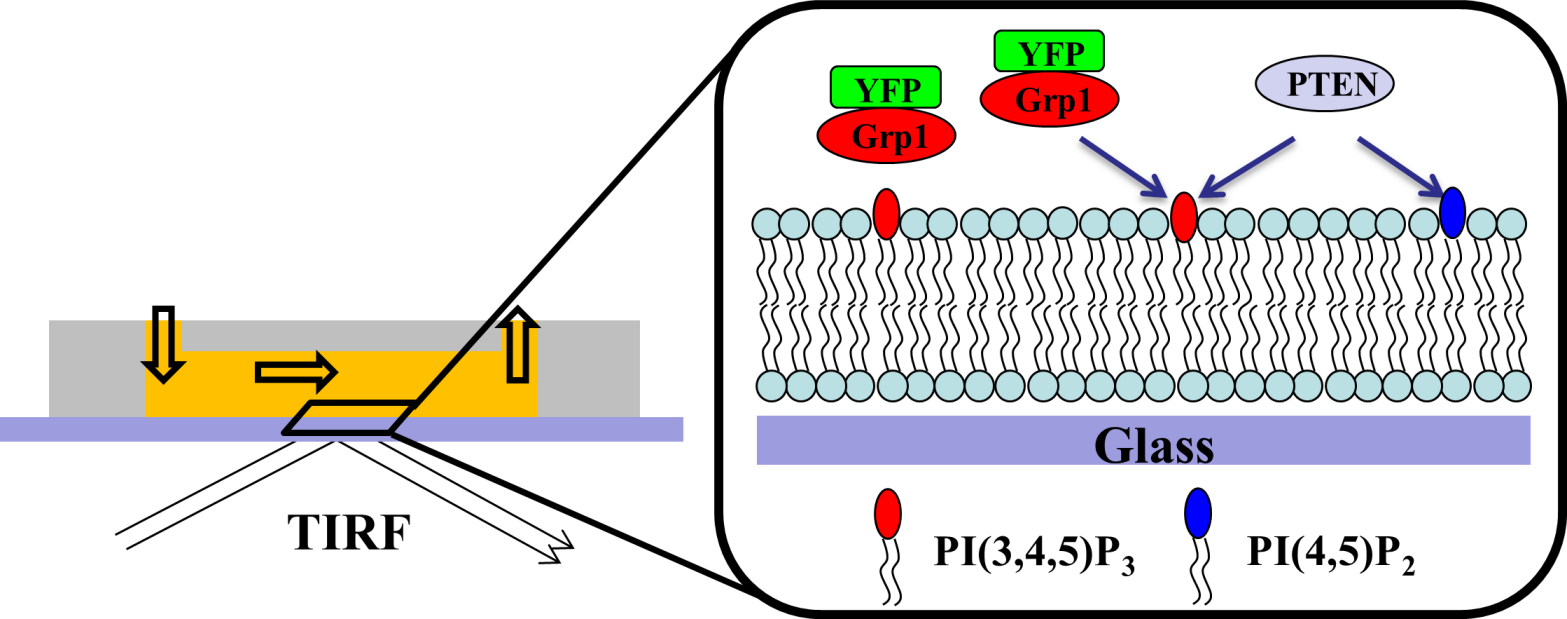
Interfacial protein binding and enzyme catalysis on supported lipid bilayer in flow chamber. Schematic illustration of the flow chamber / TIRF detection system. Small unilamellar vesicles (SUV) are injected to form a supported lipid bilayer on the glass surface. Subsequently, YFP-PHGrp1 and PTEN / YFP-PHGrp1 are injected sequentially into the measurement chamber, and the YFP-PHGrp1 signal change on the membrane is monitored by TIRF microscopy imaging.

In order to quantitatively study PTEN mediated PI(3,4,5)P3 hydrolysis, we used a fluorescence approach based on solid supported lipid bilayer (SSB) membranes in a flow chamber (Fig 1). SSBs were produced via injection and subsequent fusion of PI(3,4,5)P3-containing small unilamellar vesicles (SUV) to a microscope cover slide which sealed an Ibidi flow chamber. All of the lipid bilayers used in this study consisted of a DOPC (dioleoylphosphatiylcholine) background, and also contained 5% dioleoylphosphatidylserine (DOPS, which increases PTEN binding [11]) and small amounts of phosphoinositide lipids, as well as the lipid fluorophore TR-DHPE (Texas-Red-dihexadecanoylphosphatidylethanolamine). Here, and later in the manuscript, lipid compositions are always referred to as mol%.

Before investigating PTEN-mediated phosphoinositide conversion by means of a biosensor approach, the sensor itself has to be characterized. To study the biosensor / membrane interaction, the PI(3,4,5)P3 sensor YFP-PHGrp1 was injected into the measurement chamber, and its signal change on the membrane surface was monitored through total internal reflection fluorescence microscopy (TIRF) imaging. An equivalent approach was used further below to monitor PTEN-mediated PI(3,4,5)P3 hydrolysis kinetics. Buffer exchange was used to initiate YFP-PHGrp1 association and dissociation kinetics during continuous flow at a flow velocity of 1 cm/s, while PTEN / YFP-PHGrp1 was injected to observe PI(3,4,5)P3 hydrolysis by PTEN. YFP-PHGrp1 was co-injected with PTEN to avoid the spontaneous dissociation of membrane-bound YFP-PHGrp1. We determined the deadtime of the flow chamber for a series of flow velocity (Figure A in S2 File). At a flow velocity of 1 cm/s, we found a dead time of about 2 s, which is substantially shorter than the time frame over which relevant membrane mediated kinetics occurred (see below).

**Fig 2:**
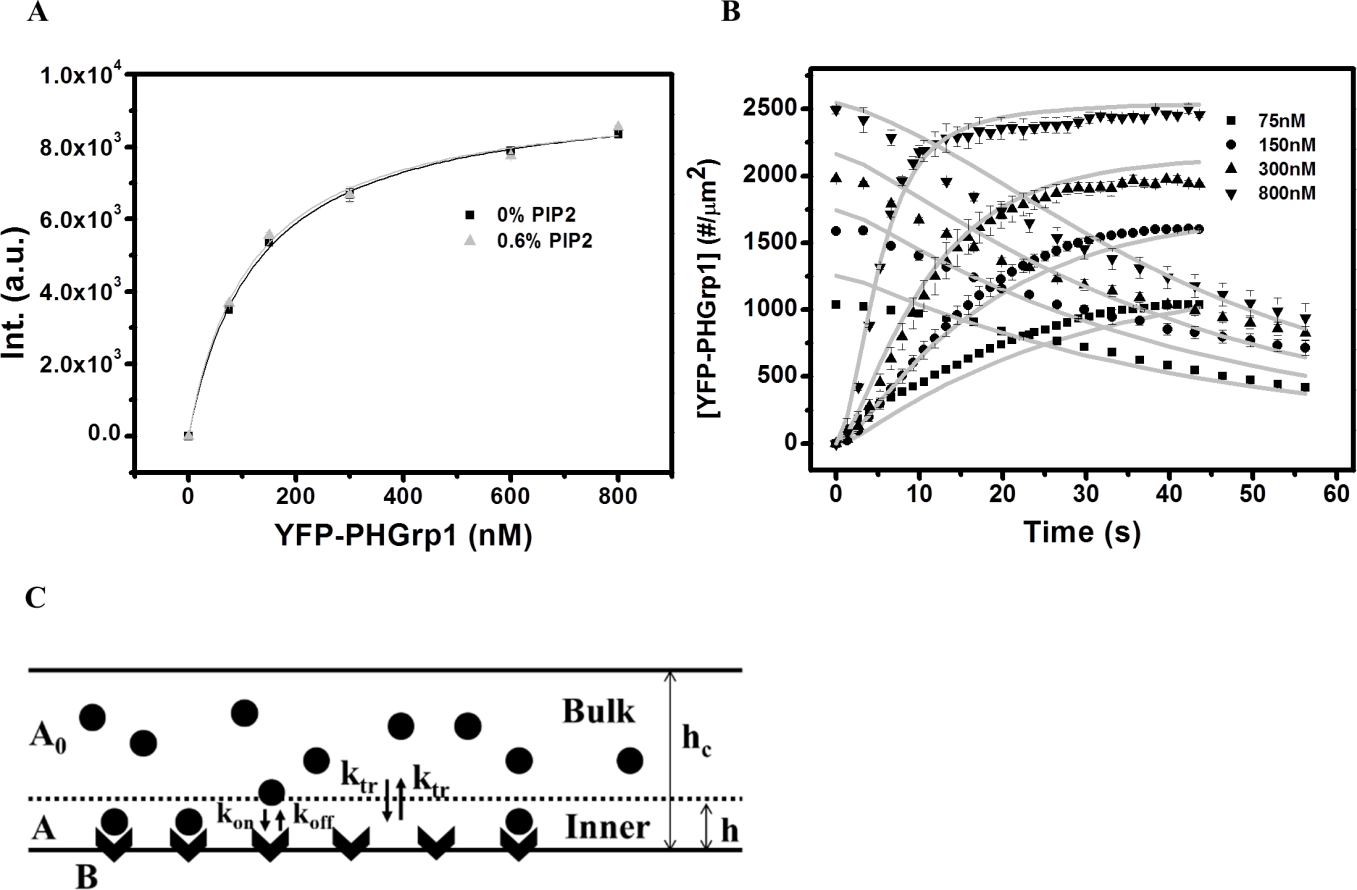
Association and dissociation kinetics of YFP-PHGrp1. (A) Binding isotherm of YFP-PHGrp1 on 0.2% PI(3,4,5)P3 / 5% DOPS / 94.6% DOPC / 0.2% TR-DHPE (square, with $I_{max}^{R}$ = 9612.30±132.42 (a.u) and *K*_d_ = 126.51±6.07 nM) and 0.6% PI(4,5)P2 / 0.2% PI(3,4,5)P3 / 5% DOPS / 94% DOPC / 0.2% TR-DHPE (triangle, with $I_{max}^{R}$ = 9526.64±265.70 (a.u) and *K*_d_ = 116.91±11.74 nM). Data points are mean ± standard errors of the mean (SEM) for N = 3 measurements. The uncertainty of fit parameters *K*_d_ and $I_{max}^{R}$ is standard deviation. The solid lines represent the best fit of experimental data using Langmuir isotherm formula $\frac{I_{max}^{R}[P]}{K_{d}+[P]}$. (B) YFP-PHGrp1 association and dissociation curves at 0.2% PI(3,4,5)P3 / 5% DOPS / 94.6% DOPC / 0.2% TR-DHPE membrane. The y-axis represents the surface concentration of YFP-PH-Grp1. The bulk concentrations of YFP-PHGrp1 for association kinetics measurements are 75nM (square), 150nM (circle), 300nM (triangle), 800nM (down-pointing triangle), respectively. After the signals reached their plateau values, buffer was injected to initiate YFP-PHGrp1 dissociation kinetics. The data were globally fitted with a compartment model (Fig 2C, Eqs 1 and 2), the fitting curves are shown as grey lines. The value of the fitting parameters are $k_{a}^{Grp1}$ = 1.68±0.18 μM^-1^s^-1^, $k_{d}^{Grp1}$ = 0.16±0.02 s^-1^, *k*_tr_ = (1.50±0.20)*10^−6^ m/s, and *h* = 17.83±2.23 μm. Data points are mean ± SEM (typically *N*≧3, but always *N*≧2 measurements). The uncertainty of fit parameters is standard deviation. (C) Schematic illustration of the compartment model. The bulk solution and inner compartment are separated by the dotted line.

We first sought to determine the surface density of YFP-PHGrp1 on a supported lipid bilayer and to exclude the possibility that PI(4,5)P2 affects YFP-PHGrp1 binding under our experimental conditions. For these purposes, we obtained YFP-PHGrp1 binding isotherms among bilayers containing 0% and 0.6% PI(4,5)P2, respectively (Fig 2A). Here we used 0.6% PI(4,5)P2 because it refers to the maximal PI(4,5)P2 content of membranes used for PTEN-mediated hydrolysis experiments described further below. Binding data were fitted with the Langmuir adsorption isotherm: $I_{max}^{R}[P]/(K_{d}+[P])$. Here, *K*_d_ is the equilibrium dissociation constant and [*P*] is the bulk concentration of protein. $I_{max}^{R}$ is the fluorescence intensity at surface saturation and is expressed in arbitrary units of the detector signal using the same gain setting for each isotherm. We obtained identical (within experimental uncertainties) $I_{max}^{R}$ and *K*_d_ values for YFP-PHGrp1 on both membrane types (For 0% PI(4,5)P2: $I_{max}^{R}$ = 9612.30±132.42 (a.u) and *K*_d_ = 126.51±6.07 nM; 0.6% PI(4,5)P2: $I_{max}^{R}$ = 9526.64±265.70 (a.u) and *K*_d_ = 116.91±11.74 nM). This agrees with literature findings showing that PH-Grp1 has high specificity towards PI(3,4,5)P3 over PI(4,5)P2 [16, 33]. These findings justify the exclusion of the influence of PI(4,5)P2 content on YFP-PHGrp1 membrane binding. The binding isotherm is also used to determine the surface concentration of YFP-PHGrp1 throughout the paper (details of the calculation are in the S1 File).

We then describe the association and dissociation kinetics of YFP-PHGrp1 on the membrane (Fig 2B). Solutions with different concentrations of YFP-PHGrp1 were injected in order to measure association kinetics. Buffer was subsequently injected at a flow velocity of 1 cm/s to study dissociation kinetics. We fitted the data with a kinetic compartment model (Fig 2C), which accounts for the presence of a depletion layer near the membrane surface [34–36]. The compartment model considers the fact that the binding kinetics of ligands to a membrane receptor depends on the kinetics of transport through the bulk solution. The depletion layer near the binding surface in a microfluidic channel device is also called the inner compartment of a channel. Distinguished from the depletion layer is the bulk solution, also called the outer compartment, where the concentration of ligand remains identical to the initial concentration of the injected solution.

The reaction scheme of the compartment model is reviewed below, along with the corresponding differential equations; Eqs 1 and 2:

$$A+B\overset{k_{on}}{\underset{k_{off}}{⇄}}AB\;A_{0}\overset{k_{tr}}{\underset{k_{tr}}{⇄}}A$$

$$\frac{\partial [AB]}{\partial t}=k_{on}[A][B]-k_{off}[AB]$$ (1)

$$\frac{\partial [A]}{\partial t}=\frac{1}{h}\{k_{tr}({\left[A\right]}_{0}-[A])+k_{off}[AB]-k_{on}[A][B]\}$$ (2)

*A*_0_ and *A* (units: μM) are the concentration of ligands (here YFP-PHGrp1) in the bulk solution (outer compartment) and in the inner compartment, respectively, while *B* (unit: μM m) is the surface receptor (here PI(3,4,5)P3) concentration within the supported lipid bilayer. *AB* (unit: μM m) is the surface concentration of protein bound to the lipid (here PI(3,4,5)P3-bound YFP-PHGrp1). The unit of surface concentration we used in this manuscript is μM m, which can be easily converted to commonly used unit number of molecules per micrometer square (1 μM m = 6*10^8^ molecules / μm^2^). The association constant is *k*_on_ (unit: μM^-1^ s^-1^), the dissociation constant is *k*_off_ (unit: s^-1^), *k*_tr_ (unit: m/s) is the transport coefficient of ligands between inner and outer compartment, and *h* (unit: μm) is the height of the inner compartment.

We determined an association constant $k_{a}^{Grp1}$ for YFP-PHGrp1 of 1.68±0.18 μM^-1^ s^-1^ and a dissociation constant $k_{d}^{Grp1}$ of 0.16±0.02 s^-1^. These values are close to previously determined values measured by stopped flow on PC / Dansyl-PE / PIP3 (92 / 5 / 3) membranes [37]. Although in the literature the membrane contains a higher concentration of PI(3,4,5)P3, we would only expect it to change the observed binding / unbinding rates but not the rate constants *k*_on_ and *k*_off_. More details about the binding / unbinding rates are discussed in the S4 File. The transport coefficient can be expressed as follows [34]:

$$k_{tr}∼0.855*{\left(\frac{v_{c}D^{2}}{h_{c}x}\right)}^{1/3}$$ (2A)

where *D* is the diffusion coefficient, *v*_c_ is the linear flow velocity, *h*_c_ is the height of the flow chamber, and *x* is the distance of the observation point from the inlet (*D* = 100 μm^2^/s, *v*_c_ = 1 cm/s, *h*_c_ = 515 μm, *x* = 1 cm in our system). The transport coefficient calculated based on this formula is 2.3*10^−6^ m/s, which is close to our fitted value of 1.5*10^−6^ m/s (see Table 1). The height of the inner compartment can be estimated by the thickness of the Nernst boundary layer, which can be expressed as follows [38]:

$${\delta_{N}∼D}^{\frac{1}{3}}u^{\frac{1}{6}}{\left(\frac{x}{v_{c}}\right)}^{1/2}$$ (2B)

where *u* is the kinematic viscosity (*u* = 10^−6^ m^2^/s; *D*, *v*_c_, and *x* are the same as above). The thickness of the calculated boundary layer is 46 μm, which is of the same order of magnitude as the fitted value of 18 μm (see Table 1).

**Table 1:** Value of fitting parameters obtained for fits to data shown in Figs 2B, 5 and 6A.

	Parameters	Values	Literature value
$k_{a}^{Grp1}$	**Association rate of Grp1 to the lipid bilayer**	**1.7±0.2 μM^-1^ s^-1^**	2.95 μM^-1^ s^-1^ (ref [37])*
$k_{d}^{Grp1}$	**Dissociation rate of Grp1 from the lipid bilayer**	**0.16±0.02 s^-1^**	**0.28 s^-1^ (ref [37])**
***k*_tr_**	**Transport coefficient**	(1.5±0.2)*10^−6^ m/s	2.3*10^−6^ m/s (calculation) **
***h***	**Height of inner compartment**	**18±2 μm**	46 μm (calculation)**
$k_{cat}^{PTEN}$	**Dephosphorylation rate of PI(3,4,5)P3 by PTEN**	**11±1 s^-1^**	**15 s^-1^ (ref [13]); 0.5 s^-1^ (ref [12])**
$k_{a}^{PTEN}$	**Association rate of PTEN to lipid bilayer**	(1.7±0.3)*10^−3^ m/s; 0.71 μM^-1^ s^-1^*	**0.5 μM^-1^ s^-1^ (ref [42])**
$k_{d}^{PTEN}$	**Dissociation rate of PTEN from lipid bilayer**	**0.7±0.1 s^-1^**	**1 s^-1^ (ref [13]); 3–7.7 s^-1^ (ref [28])**
$k_{a}^{PTEN-PI(4,5)P2}$	**Association rate of PTEN to PI(4,5)P2**	**200±22 μM^-1^ s^-1^**	**50 μM^-1^ s^-1^ (ref [12], assumed)**
$k_{d}^{PTEN-PI(4,5)P2}$	**Dissociation rate of PTEN from PI(4,5)P2**	**0.30±0.05 s^-1^**	**1 s^-1^ (ref [13]); 0.1 s^-1^ (ref [12], assumed)**
$k_{cat}^{PTEN-PI(4,5)P2}$	**Dephosphorylation rate of PI(3,4,5)P3 by PTEN-PI(4,5)P2**	**19±2 s^-1^**	****
$K_{M}^{PTEN}$	**Michaelis constant of PTEN dephosphorylation reaction**	(2.0±0.3)*10^−3^ μM m	****
$K_{M}^{PTEN-PI(4,5)P2}$	**Michaelis constant of PTEN-PI(4,5)P2 dephosphorylation reaction**	(2.3±0.2)*10^−5^ μM m	**See S4 File (ref [43])**
***K*_PTEN,PI(4,5)P2_**	****	(7.9±0.2)*10^−6^ μM m	****
***n***	**Hill coefficient**	**2.0±0.1**	****

* Unit conversion for parameter comparison will be discussed in S4 File.

** The calculation of *k*_tr_ and *h* is shown in the main text and described by formula 2a and 2b, respectively.

The uncertainty of the fit parameters is the standard error.

### Autocatalytic reaction of PTEN hydrolysis of PI(3,4,5)P3

**Fig 3:**
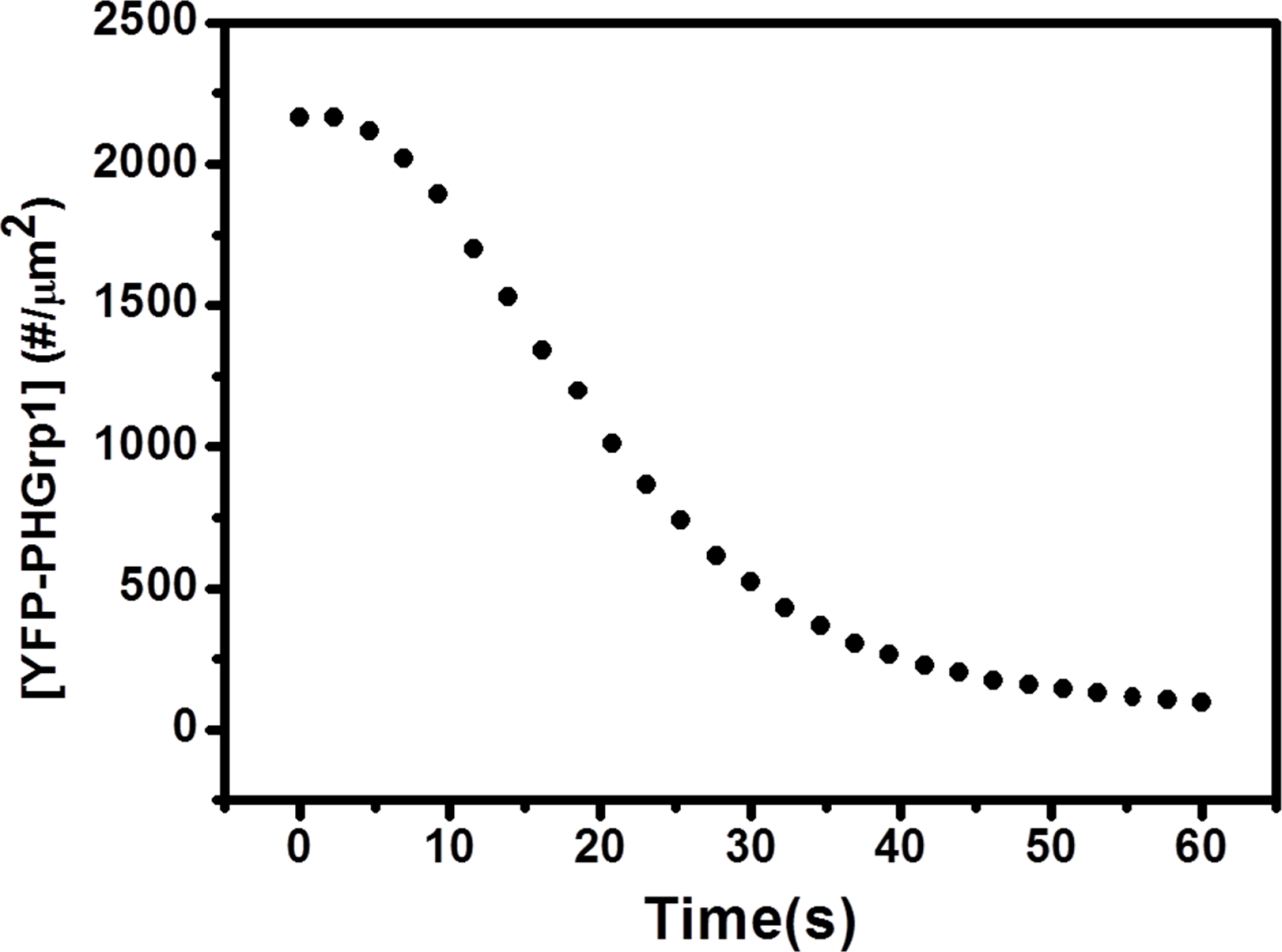
PI(3,4,5)P3 hydrolysis kinetics by PTEN on 0.2% PI(3,4,5)P3 / 0.2% PI(4,5)P2 / 5% DOPS / 94.4% DOPC / 0.2% TR-DHPE membrane. The y-axis represents the surface concentration of YFP-PHGrp1. The kinetic curve of YFP-PHGrp1 decay follows a sigmoidal shape.

Having characterized the reversible binding of YFP-PHGrp1 on our membranes, we proceeded to study PI(3,4,5)P3 hydrolysis by PTEN, under the condition of continuous flow of 1 cm/s (Figure A in S2 File). When PTEN is introduced into the measurement chamber, accessible PI(3,4,5)P3 will be hydrolyzed and thus YFP-PHGrp1 will progressively dissociate from the membrane. This process was imaged through TIRF via the YFP-PHGrp1 signal decay. We found that the YFP-PHGrp1 signal decay upon PTEN injection does not follow simple exponential decay in the presence of non-zero initial concentrations of PI(4,5)P2. Instead, sigmoidal kinetics was observed for membranes initially containing 0.2% PI(4,5)P2 (Fig 3). Qualitatively, the sigmoidal behavior implies a lag time (of about 6 s in Fig 3), after which the hydrolysis rate increases. The sigmoidally shaped kinetics curve is interesting because it is consistent with the fact that PI(3,4,5)P3 hydrolysis by PTEN is an autocatalytic reaction. This finding supports the notion that PI(4,5)P2 produced through PTEN action has positive effects on either PTEN recruitment or PTEN activation, or both.

### Theoretical model of the PTEN / Grp1 / PI(3,4,5)P3 / PI(4,5)P2 reaction system

**Fig 4:**
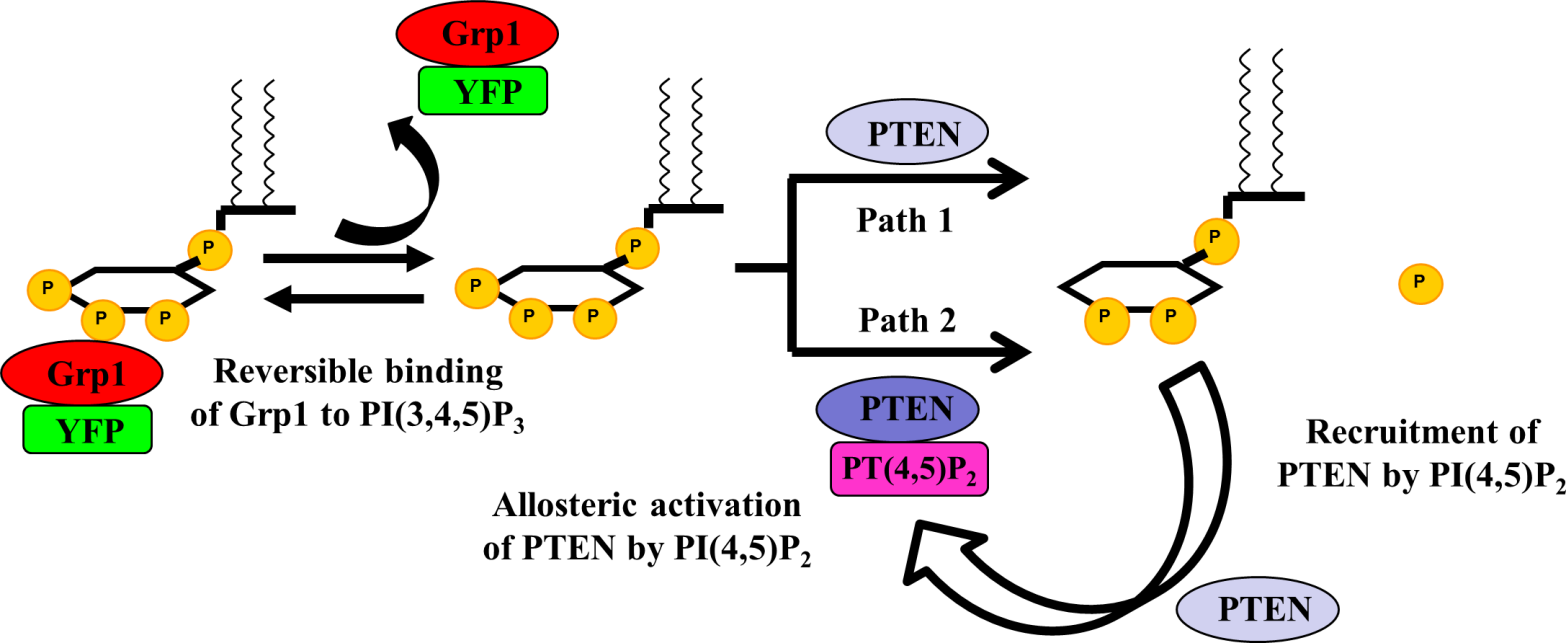
Theoretical model for PTEN Kinetics. YFP-PHGrp1 binds PI(3,4,5)P3 competitively with PTEN, which can only bind to and hydrolyze PI(3,4,5)P3 when Grp1 is not bound. We assume that free PI(3,4,5)P3 can be hydrolyzed via two paths, either by PI(4,5)P2-free PTEN (path 1, PTEN) or by PI(4,5)P2-bound PTEN (path 2, PTEN-PI(4,5)P2). When bound to PI(4,5)P2, PTEN can be allosterically activated and increases its hydrolysis activity towards PI(3,4,5)P3. Newly formed PI(4,5)P2 can also recruit more PTEN from solution to the membrane.

On the basis of the positive effect of PI(4,5)P2 on PTEN hydrolysis of PI(3,4,5)P3, we sought kinetic models capable of suitably describing this autocatalytic reaction (Fig 4). In our model, we consider the reversible binding of YFP-PHGrp1 to PI(3,4,5)P3. We assume that PI(3,4,5)P3 cannot be hydrolyzed by PTEN while bound to Grp1, as both the catalytic site of PTEN and the PH domain of Grp1 bind to the inositol ring of PI(3,4,5)P3 [4, 39]. The phosphoinositide sensor YFP-PHGrp1 therefore does not merely act as a reporter for PI(3,4,5)P3 hydrolysis, but also competes with PTEN in binding to PI(3,4,5)P3. When YFP-PHGrp1 dissociates from PI(3,4,5)P3, PTEN can bind to and dephosphorylate PI(3,4,5)P3 to yield PI(4,5)P2. The newly formed PI(4,5)P2 can recruit more PTEN (via its PI(4,5)P2 binding domain) to the membrane, which accelerates the PI(3,4,5)P3 hydrolysis due to PTEN enrichment on the membrane. Furthermore, PTEN can be allosterically activated when bound to PI(4,5)P2, which increases its phosphatase activity toward PI(3,4,5)P3. In our model, we assume that PI(3,4,5)P3 can be hydrolyzed by both membrane-bound PTEN devoid of PI(4,5)P2 (PTEN) and by PI(4,5)P2-bound PTEN (PTEN-PI(4,5)P2). To account for PI(4,5)P2 binding leading to allosteric activation of PTEN and thereby increasing its phosphatase activity, we defined an effective catalysis rate with the help of the Hill equation [40, 41] (Eq 6 in S3 File). The reaction equilibria are listed below.

$$Grp1_{comp\_sol}+[PI(3,4,5)P3]\overset{k_{a}^{Grp1}}{\underset{k_{d}^{Grp1}}{⇄}}{\left[Grp1-PI(3,4,5)P3\right]}_{m}$$ (3)

$$PTEN_{comp\_sol}\overset{k_{a}^{PTEN}}{\underset{k_{d}^{PTEN}}{⇄}}PTEN_{m}$$ (4)

$$PTEN_{comp\_sol}+{\left[PI(4,5)P2\right]}_{m}\overset{k_{a}^{PTEN-PI(4,5)P2}}{\underset{k_{d}^{PTEN-PI(4,5)P2}}{⇄}}{\left[PTEN-PI(4,5)P2\right]}_{m}$$ (5)

$$PTEN_{m}+{\left[PI(3,4,5)P3\right]}_{m}\overset{k_{cat}^{PTEN}}{\to }PTEN_{m}+{\left[PI(4,5)P2\right]}_{m}$$ (6)

$${\left[PTEN-PI(4,5)P2\right]}_{m}+{\left[PI(3,4,5)P3\right]}_{m}\overset{k_{cat}^{PTEN-PI(4,5)P2}}{\to }{\left[PTEN-PI(4,5)P2\right]}_{m}+{\left[PI(4,5)P2\right]}_{m}$$ (7)

The differential equations corresponding to the above reaction equilibria are listed in the S3 File. Reaction 3 describes the reversible binding of YFP-PHGrp1 to PI(3,4,5)P3. PI(4,5)P2-independent and PI(4,5)P2-dependent association of solution PTEN to the membrane are described by reactions 4 and 5, respectively. Two routes of PI(3,4,5)P3 hydrolysis are then considered. In one way, PI(3,4,5)P3 is hydrolyzed by PI(4,5)P2-free PTEN, described by reaction 6. In the second way, PI(3,4,5)P3 is hydrolyzed by PI(4,5)P2-bound PTEN, described by reaction 7.

### Recruitment of PTEN by PI(4,5)P2 occurs in combination with PTEN-PI(4,5)P2 allosteric activation to produce autocatalytic PI(3,4,5)P3 hydrolysis

**Fig 5:**
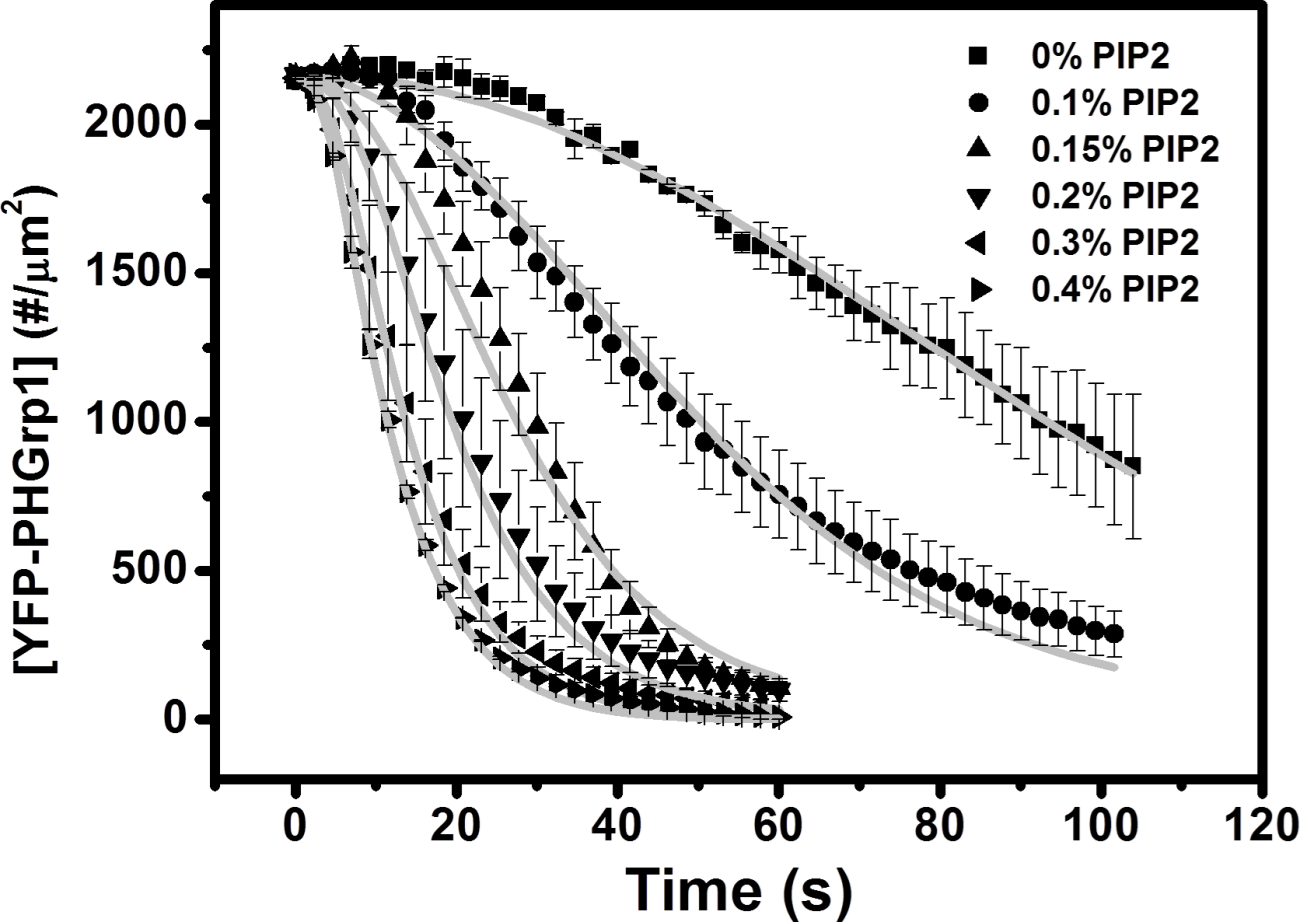
PI(3,4,5)P3 hydrolysis kinetics by PTEN at DOPC / DOPS membrane with different % of PI(4,5)P2. The membrane composition: 0.2% PI(3,4,5)P3 + 5% DOPS + n% PI(4,5)P2 + (94.6-n)% DOPC + 0.2% TR-DHPE. n = 0 (square), n = 0.1 (circle), n = 0.15 (triangle), n = 0.2 (down-pointing triangle), n = 0.3 (left-pointing triangle), n = 0.4 (right-pointing triangle). The y-axis represents the surface concentration of YFP-PHGrp1. The bulk concentration of YFP-PHGrp1 is 300 nM and the bulk concentration of PTEN is 100 nM. The global fitting curves based on our kinetic PTEN catalysis model (Fig 4, Eqs 1–7 in S3 File) are shown as a grey solid line. The rate of YFP-PHGrp1 signal decay and thus PTEN action increases with initial PI(4,5)P2 surface concentration. Data points are mean ± SEM (typically *N*≧3, but always *N*≧2 measurements).

To further evaluate the autocatalytic nature PI(3,4,5)P3 hydrolysis by PTEN, we varied the concentration of PI(4,5)P2 within DOPS / DOPC mixtures and studied how the surface concentration YFP-PHGrp1 changes with time after PTEN injection (Fig 5). Clearly, the PI(3,4,5)P3 hydrolysis rate increases with increasing initial PI(4,5)P2 content in the membrane.

**Fig 6:**
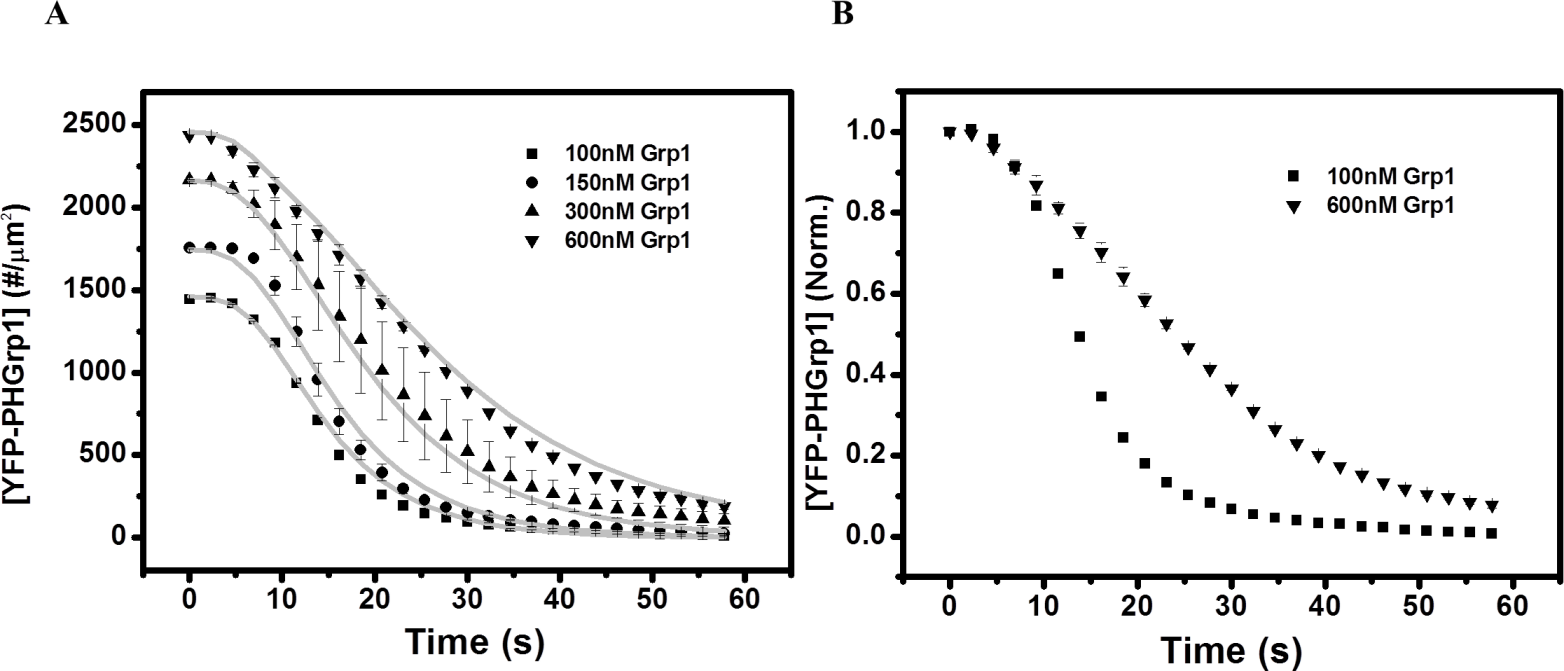
Kinetics of PI(3,4,5)P3 hydrolysis by PTEN at different concentrations of YFP-PHGrp1. (A) The membrane composition is 0.2% PI(3,4,5)P3 + 5% DOPS + 0.2% PI(4,5)P2 + 94.4% DOPC + 0.2% TR-DHPE and the bulk concentration of YFP-PHGrp1 is 100 nM (square), 150 nM (circle), 300 nM (triangle), and 600 nM (down-pointing triangle), respectively. The bulk concentration of PTEN is 100 nM. The grey solid lines are the global fitting results based on our kinetic PTEN catalysis model (Fig 4, Eqs 1–7 in S3 File). Data points are mean ± SEM (typically *N*≧3, but always *N*≧2 measurements). (B) Normalized kinetic traces of PI(3,4,5)P3 hydrolysis by PTEN at 100 nM (square) and 600 nM (down-pointing triangle) of YFP-PHGrp1. The rate of YFP-PHGrp1 signal decay upon PTEN injection increases with decreasing bulk YFP-PHGrp1 concentration, which agrees with the model that YFP-PHGrp1 competitively binds to PI(3,4,5)P3 with PTEN.

In addition, we evaluated the effect of YFP-PHGrp1 on PI(3,4,5)P3 hydrolysis by varying the bulk concentration of YFP-PHGrp1 systematically (Fig 6A). The PI(3,4,5)P3 hydrolysis rate is observed to decrease with increasing YFP-PHGrp1 bulk concentration (Fig 6B). The slower kinetics at high concentration of YFP-PHGrp1 can be rationalized by the fact that competitive binding of Grp1 to PI(3,4,5)P3 reduces the probability of PTEN accessing its substrate.

The global fitting result of PI(3,4,5)P3 hydrolysis by PTEN at different surface concentrations of PI(4,5)P2 and different YFP-PHGrp1 bulk concentrations is shown as grey lines in Figs 5 and 6. The corresponding fitting parameters are listed in Table 1.

**Fig 7:**
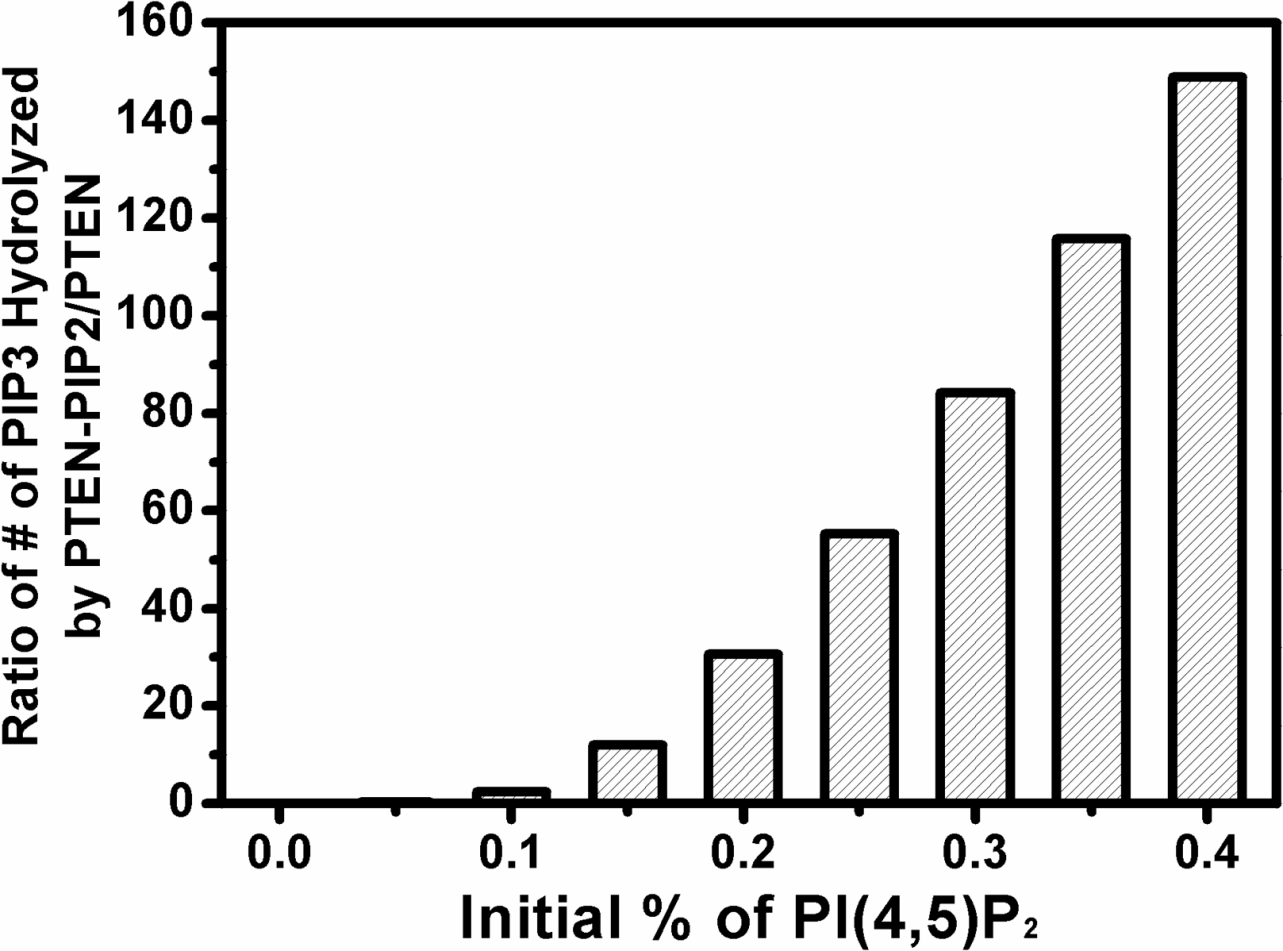
Simulation of PI(3,4,5)P3 hydrolysis by PTEN-PI(4,5)P2 and PTEN. Based on the theoretical model we proposed (Fig 4) and the fitting parameters it yielded (Table 1), we examined how the ratio of the number of PI(3,4,5)P3 molecules hydrolyzed by PTEN-PI(4,5)P2 versus PTEN increased with rising PI(4,5)P2 concentration in the supported lipid bilayer.

Based on our model, PI(3,4,5)P3 can be hydrolyzed by two different forms of PTEN: PI(4,5)P2-free PTEN (path 1, PTEN) and PI(4,5)P2-bound PTEN (path 2, PTEN-PI(4,5)P2, shown in Fig 4). The next question we would like to answer is which path contributes more to PI(3,4,5)P3 hydrolysis. We determined the ratio of PI(3,4,5)P3 hydrolysis contributed by PTEN and PTEN-PI(4,5)P2 at different initial surface concentrations of PI(4,5)P2 (Fig 7). By incorporating fitting parameters (Table 1) into our PTEN model, we found that the ratio of the number of PI(3,4,5)P3 molecules hydrolyzed by PTEN-PI(4,5)P2 to those hydrolyzed by PTEN increases with greater initial % of PI(4,5)P2. In other words, which reaction path dominates PI(3,4,5)P3 hydrolysis is determined by the initial PI(4,5)P2 concentration of the membrane. When there was no initial PI(4,5)P2 in the bilayer, path 1 was dominant in PI(3,4,5)P3 hydrolysis. Path 2 became comparable to path 1 at initial 0.1% PI(4,5)P2, and became dominant when the initial PI(4,5)P2 increased to 0.15%. The routes of PI(3,4,5)P3 hydrolysis switched from path 1 to path 2 when initial PI(4,5)P2 surface concentration was increased from 0% to 0.15%.

**Fig 8:**
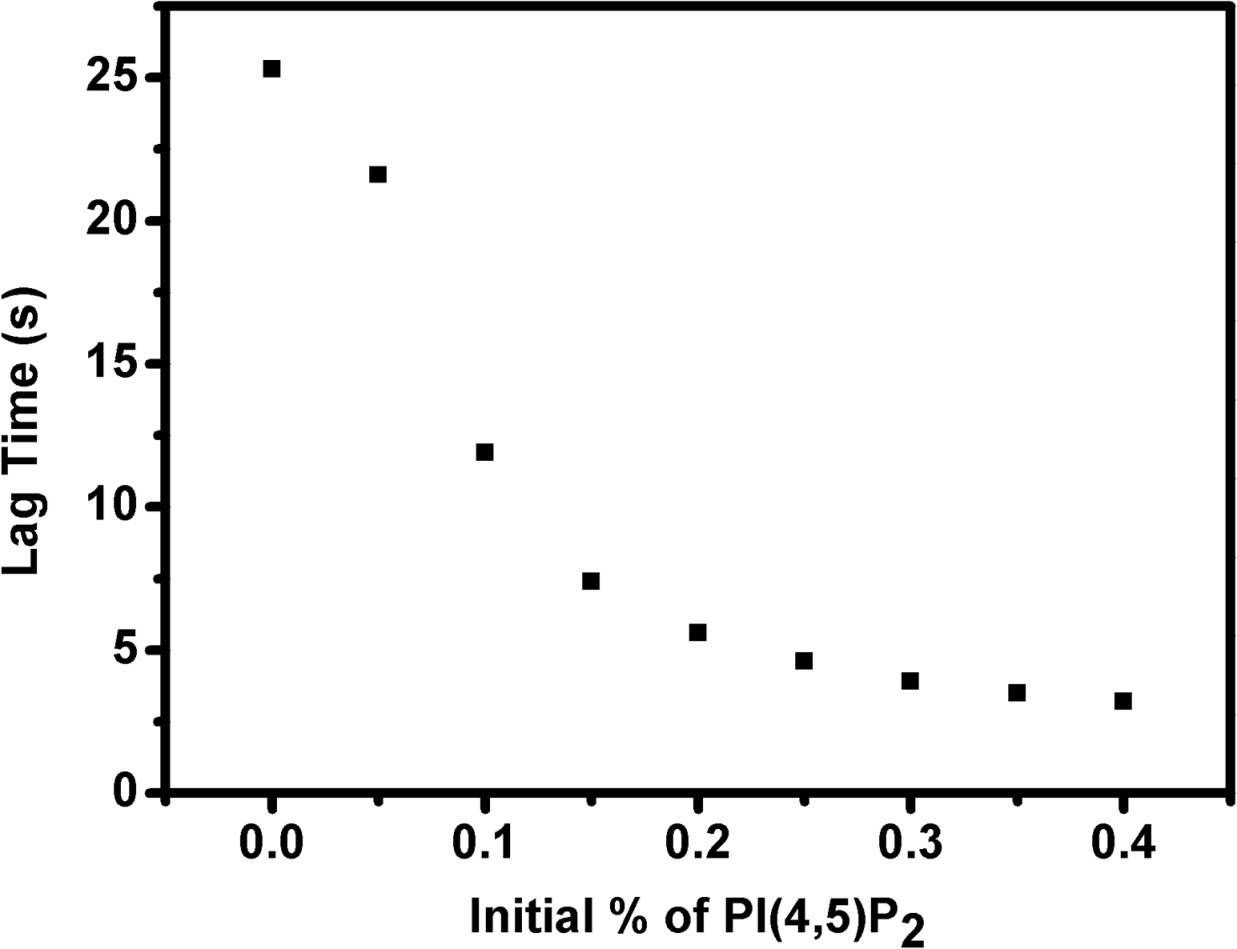
Simulation of change of lag time of PI(3,4,5)P3 hydrolysis with initial % of PI(4,5)P2. Based on the theoretical model we proposed (Fig 4) and the fitting parameters it yielded (Table 1), we predicted the lag time for YFP-PHGrp1 signal decay after PTEN injection. We defined the lag time as the time needed for the YFP-PHGrp1 signal to decay to 95% of its initial value. Clearly, the lag time shortens with increasing initial PI(4,5)P2% on the membrane.

We also asked the question in what form the lag time of PI(3,4,5)P3 hydrolysis changes with initial % of PI(4,5)P2. We defined the lag time as the reaction time needed for the YFP-PHGrp1 signal to decay to 95% of its initial value. In Fig 8, we show that the lag time decreases with increasing initial PI(4,5)P2 content. When the initial concentration of PI(4,5)P2 in the membrane is low, it takes more time for PTEN to bind the membrane and dephosphorylate PI(3,4,5)P3.

**Fig 9:**
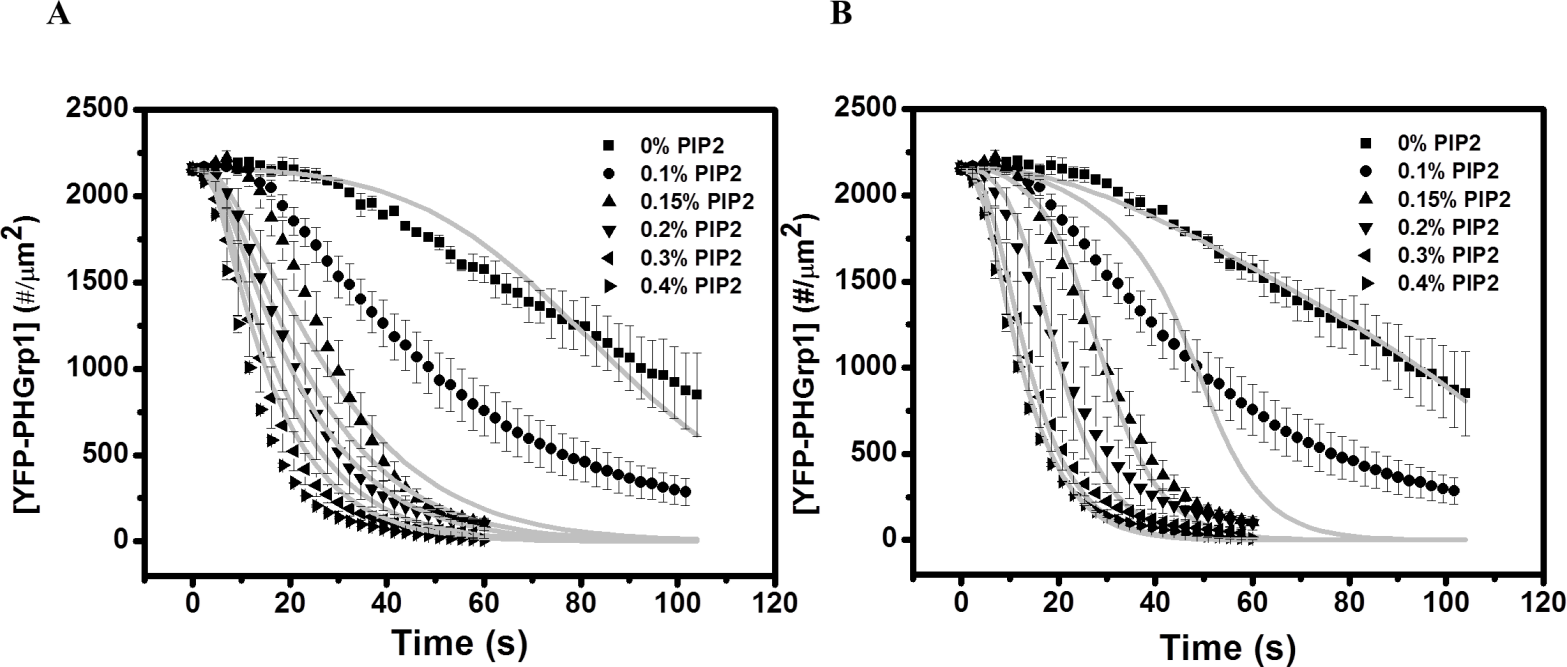
PI(3,4,5)P3 hydrolysis kinetics by PTEN at DOPC / DOPS membrane with different % of PI(4,5)P2. The membrane composition: 0.2% PI(3,4,5)P3 + 5% DOPS + n% PI(4,5)P2 + (94.6-n)% DOPC + 0.2% TR-DHPE. n = 0 (square), n = 0.1 (circle), n = 0.15 (triangle), n = 0.2 (down-pointing triangle), n = 0.3 (left-pointing triangle), n = 0.4 (right-pointing triangle). Data points are mean ± SEM (typically *N*≧3, but always *N*≧2 measurements). **(A)** Fit it with recruitment only model (Eqs 1–5, 10, 11 in S3 File) **(B)** Fit it with allosteric activation only model (Eqs 1–4, 8, 9 in S3 File).

To answer the question whether the model we proposed can be viewed as a minimum kinetic model describing PI(3,4,5)P3 hydrolysis by PTEN, we tested two simplified models: 1) a model assuming PI(4,5)P2 induced recruitment of PTEN only, and 2) a model assuming PI(4,5)P2 mediated allosteric activation of PTEN only. In the “recruitment only” model, PTEN and PTEN-PI(4,5)P2 are assumed to have the same turnover number *k*_cat_ and interfacial Michaelis-Menten constant *K*_M_ of PI(3,4,5)P3. In other words, PI(4,5)P2 affects membrane binding, but does not affect catalytic properties of PTEN at the molecular level. Therefore, the Hill equation was not included in this model. In the “allosteric activation only” model, PI(4,5)P2 is assumed to be unable to promote PTEN membrane binding, but able to allosterically activate PTEN. We fitted PI(3,4,5)P3 hydrolysis kinetics curves for different PI(4,5)P2 surface concentrations as well as different YFP-PHGrp1 bulk concentrations simultaneously with both models (Fig 9). From the fitting results we noticed that though the individual “recruitment only” model and “allosteric activation only” model fit some curves well, it can’t globally fit all curves. Some fitting curves deviate dramatically from the kinetic curves. The results clearly demonstrate that neither one of these two models are sufficient to fit the data, strongly suggesting that the positive effect of PI(4,5)P2 on PTEN hydrolysis of PI(3,4,5)P3 results from a combination of both recruitment and allosteric activation effect.

## Discussion

Several reports indicate that the rate of PTEN-catalyzed PI(3,4,5)P3 hydrolysis is accelerated by its own product PI(4,5)P2 [10, 43, 44]. However, two main mechanisms explaining the autocatalytic nature of PI(3,4,5)P3 dephosphorylation by PTEN are proposed. The Downes [43] lab proposed that the newly-formed PI(4,5)P2 on the membrane interacts with the N-terminal PI(4,5)P2-binding domain of PTEN, which increases the binding of PTEN to the membrane. On the other hand, the Ross [10] lab proposed that the origin of autocatalysis comes from allosteric activation. This mechanism is supported by IR spectroscopy measurements, which show a change of PTEN conformation when PI(4,5)P2 binds to the N-terminus of PTEN [45]. Whether both positive regulations of PTEN by PI(4,5)P2 exist is one of the key questions answered in this work. On the other hand, there are many signaling proteins with PH domains that specifically bind to PI(3,4,5)P3 and that are involved in the PI3K signaling pathway [14]. How PI(3,4,5)P3-binding proteins affect the PI(3,4,5)P3 hydrolysis by PTEN is another issue to be resolved. The YFP-PHGrp1 not only acts as a PI(3,4,5)P3 sensor but is also used to study how PI(3,4,5)P3-binding proteins affect the rate of PTEN dephosphorylation of PI(3,4,5)P3.

Our findings first confirm that the reaction catalyzed by PTEN is autocatalytic by showing a sigmoidal shape for PI(3,4,5)P3 hydrolysis (Fig 3). We then varied the initial concentration of PI(4,5)P2 in the membrane and found that the rate of PI(3,4,5)P3 hydrolysis increases significantly with increasing PI(4,5)P2 concentration (Fig 5), which further supports the notion that PI(4,5)P2 positively regulates PTEN action (Fig 3). The concentration of PHGrp1 is another factor that affects PI(3,4,5)P3 hydrolysis by PTEN. The rate of hydrolysis is attenuated with increasing concentration of Grp1. To study the origin of PTEN-PI(4,5)P2 autocatalysis and the effect of PHGrp1 concentration on PI(3,4,5)P3 dephosphorylation, we considered several kinetic models to fit the curves. A recruitment-only model and an allosteric-activation-only model could not successfully fit the kinetic traces of PI(3,4,5)P3 hydrolysis. A model combining both mechanisms is needed to fit the curves satisfactorily, and a plethora of kinetic parameters are obtained for this YFP-PHGrp1 / PTEN / bilayer system, including the interfacial Michaelis-Menten constant and a Hill coefficient. Our kinetic model allows us to discern the relative contributions of two reaction paths of PI(3,4,5)P3 hydrolysis by PTEN as modulated by the PI(4,5)P2 content of the membrane. Path 1 (PI(4,5)P2-free PTEN) dominates at low initial PI(4,5)P2 concentration (<0.15%), while path 2 (PI(4,5)P2-bound PTEN) dominates at relatively high initial PI(4,5)P2 concentration (≧0.15%). The lower rate of PI(3,4,5)P3 hydrolysis at higher PHGrp1 concentrations can be explained by the competitive binding of PTEN and Grp1 to PI(3,4,5)P3.

The values of fitting parameters are comparable to the literature values. The Michaelis-Menten constant of PTEN and PTEN-PI(4,5)P2 are 2.03*10^−3^ μM m (1.22*10^6^ molecules/μm^2^) and 2.30*10^−5^ μM m (1.38*10^4^ molecules/μm^2^), respectively. To compare the enzyme kinetic efficiency of PTEN with previous published PTEN results, we calculate the specificity constant *k*_cat_/*K*_M_ of PTEN and PTEN-PI(4,5)P2 as 792.7 X_s_^-1^ min^-1^, and 115585.5 X_s_^-1^ min^-1^, respectively (X_s_: mole fraction). McConnachie et al. measured a specificity constant of PTEN equal to 182500 Xs^-1^ min^-1^ through surface-dilution methods [43], which is comparable to the PI(4,5)P2-bound PTEN in our model. Other parameters need to be converted for comparison due to different models and measurement methods used, which are shown in the S4 File. The correlation of fit parameters is a concern that we may need to consider when determining unique parameter estimations from the fitting [46]. There are several methods by which parameter correlation can be determined. One example is the method of *mean optimal transformations* [47]. In this method, a given parameter is systematically varied around the best fit value while refitting the remaining parameter set to enable determination of parameter correlations. Markov Chain Monte-Carlo (MCMC) method can be also used to detect parameter correlation [48–50]. However, these methods are quite complex, which renders the study of parameter correlations a challenging task for nonlinear dynamic models [46–49, 51]. The computational burden arising from repeated fitting with at least thousands of different initial guesses is another challenge, given that a single fit of our model requires on the order of an hour to be completed. For these reasons, we were forced to omit an analysis fit parameter correlations in the present project. One possible way to reduce the number of correlated parameters is through fluorescent labeling of PTEN. Though this can be potentially used to study PTEN recruitment with the goal of reducing the number of fitting parameters, it could also affect the binding properties. In fact, it has been reported that the fluorescent labeling of proteins by fluorescent proteins (or even by small synthetic molecular probes) can affect its binding behavior, including amount of protein binding and rate constants of binding [52–54]. It is for this reason that we developed a biosensor approach based on YFP-PHGrp1 to detect changes in the amount of PI(3,4,5)P3 in response to PTEN catalysis.

Our fitting model reveals (Figs 7 and 8) that small changes in the initial concentration of PI(4,5)P2 have large effects on the kinetics of PI(3,4,5)P3 hydrolysis by PTEN. This can help explain why a concentration gradient of PI(4,5)P2 and PI(3,4,5)P3 along the membrane arises in response to the stimulation by chemoattractant: a small concentration fluctuation of PI(4,5)P2 and PI(3,4,5)P3 can be amplified by the preferential binding of PTEN to transient, PI(4,5)P2-rich regions, and by subsequent allosteric activation to contribute to the amplification of PI(4,5)P2 and PI(3,4,5)P3 concentration gradients on the plasma membrane [12].

Another critical question is: how is PI(3,4,5)P3 hydrolysis impact by varying the solution concentration of PH-containing proteins? It was shown that overexpression of ARNO, a guanine nucleotide exchange factor with a specific PI(3,4,5)P3-binding PH domain, leads to broad lamellipodia development and increases cell migration [55]. The actin cytoskeleton remodeling mediated by ARNO is abolished when its PH domain is deleted, indicating proper membrane localization of ARNO requires the PH domain [55]. Venkateswarlu *et al*. further indicate that translocation of ARNO to the membrane can be blocked by the PI(4,5)P2-3 kinase (PI3K) inhibitors wortmannin and LY294002, indicating PI(3,4,5)P3 plays a critical role in ARNO recruitment to the membrane [17]. Venkateswarlu et al. further confirm that membrane translocation of ARNO is PH domain-dependent. Taken together, these results suggest that ARNO-PI(3,4,5)P3 interaction plays a critical role in actin remodeling. Our results provide a potential mechanism for prolonged PI(3,4,5)P3 signaling due to increased PH domain concentration. The increased PH domain concentration competes with PTEN for available PI(3,4,5)P3, attenuating PTEN hydrolysis of PI(3,4,5)P3.

## Materials and methods

### Protein purification

A pET30b vector containing PTEN was obtained from Addgene. The protein was expressed and purified as previously described by Ross and Gericke [11]. Briefly, PTEN was expressed in Escherichia coli BL21-(DE3) cells grown in Rich media at 37°C. Protein expression was induced with 50 μM isopropyl-β-d-thiogalactoside (IPTG) at 20°C for 21 hours. Cells were harvested by centrifugation and the pellets were resuspended in a pH = 7.4 buffer containing 500 mM NaCl, 10 mM 2-mercaptoethanol in 20 mM phosphate buffer. The cells were lysed by tip sonication and centrifuged at 4°C. The supernatant was applied to a His trap affinity column (GE, Piscataway, NJ). The protein was further purified by size exclusion and anion-exchange chromatography, and stored on ice after dithiothreitol (DTT) was added at a concentration of 10 mM. A plasmid for YFP-PHGrp1 was kindly provided by Prof. T. Balla (National Institutes of Health, NIH). The fusion protein was purified as previously described [56]. YFP-PHGrp1 was expressed in BL21-(DE3) cells and grown in Rich media at 37°C. Induction occurred with 200 μM IPTG at 18°C, and proteins were expressed for 16 hours. The fusion protein was purified by His trap affinity and size exclusion chromatography, successively.

### Small unilamellar vesicle preparation

1,2-Dioleoyl-*sn*-glycero-3-phosphocholine (DOPC), l-α-phosphatidylinositol 4,5-bisphosphate (PIP2) (brain, ammonium salt), 1,2-dioleoylphosphatidylinositol 3,4,5-trisphosphate (PIP3) (ammonium salt), 1,2-dioleoyl-*sn*-glycero-3-phospho-l-serine (DOPS) and extruder accessories were purchased from Avanti Polar Lipids (Alabaster, AL). Texas Red® 1,2-Dihexadecanoyl-*sn*-Glycero-3-Phosphoethanolamine, Triethylammonium Salt (TR-DHPE) was purchased from Invitrogen (Carlsbad, CA). Calcium- and magnesium-free 150 mM NaCl and 20 mM HEPES buffer (pH = 7.4) were used in the preparation of the vesicle dispersion. Lipid stock solutions of desired compositions were prepared in chloroform and methanol (3:1, v/v) and stored in amber glass vials to protect them from UV light. For each experiment, phospholipids were spread on the walls of a round-bottomed flask, and evacuated in a desiccator for at least two hours to produce an even lipid film. The lipid film was rehydrated, sonicated for 40 min, freeze-thawed 4 times and then extruded at room temperature 17 times through a polycarbonate filter with 50 nm pores. The resulting small unilamellar vesicles (SUVs) were stored at 4°C.

### Flow chamber fabrication and supported lipid bilayer preparation

μ-Slide VI 0.4 flow chambers were obtained from Ibidi. Glass slides were cleaned by sonicating in 2% Hellmanex solution (Hellma, Mullheim, Germany) for 30 min and then rinsed with water. Subsequently, they were further treated with NOCHROMIX (Godax Laboratories, Inc) and concentrated H_2_SO_4_ for at least 6 hours, and then extensively rinsed with water. The glass slide was plasma cleaned and attached to the Ibidi chamber with double-sided tape around, forming a closed chamber with inlet and outlet. The buffer (20 mM HEPES / 150 mM NaCl, pH = 7.4) was injected to test whether leakage occurs. Small unilamellar vesicles (SUV) were then injected and incubated for 30 min to form the supported lipid bilayer. Any excess SUVs were washed out with buffer. Finally, YFP-PHGrp1 and PTEN were injected.

### Total internal reflection fluorescence (TIRF) microscopy and flow conditions

The YFP-PHGrp1 signal on the bilayer was observed by objective-type TIRF on an inverted microscope (IX71, Olympus). The bilayer was illuminated with a 488nm laser (Coherent, Santa Clara, CA) through a 60X 1.45 NA oil objective. A 1X telescope was placed at the back focal plane of the microscope and used to adjust the incidence angle of the laser beam. Fluorescence was collected with an EM CCD (HAMAMATSU, Bridgewater, NJ) and displayed with HCImage.

In this contribution, all binding and reaction experiments were carried out under the condition of continuous flow in order to maintain a constant bulk protein concentration by preventing depletion [57, 58]. For the assessment of accurate association and dissociation rates of proteins binding and unbinding from the membrane, it is important to consider inner and outer compartment of the measurement chamber (Fig 2C) [34]. Under continuous flow, the transport coefficient can be expressed as in Eq 2A. The flow velocity of 1 cm/s was chosen for all kinetic experiments in this contribution. It resulted from a compromise between the goal to minimize dead times (Figure A in S2 File) but to also minimize protein loss during the flow channel experiments.

The fluorescence intensity of YFP-PHGrp1 on the membrane is converted to the surface concentration of YFP-PHGrp1 for quantitative analysis. The data fitting is done with MATLAB.

More details about lipid surface concentration estimation, conversion of fluorescence intensity, and data analysis methods are included in the S1 File.

## Supporting information

S1 File Lipid surface concentration estimation, conversion of fluorescence intensity, and data analysis. (PDF)

S2 File Dead time measurement. (PDF)

S3 File Differential equations of the PTEN kinetic model. (PDF)

S4 File Parameters in the PTEN kinetic model discussion. (PDF)
