# Supplementary material for: Kinetics of PTEN-mediated PI(3,4,5)P3 hydrolysis on solid supported membranes
Source: PLoS One. 2018 Feb 15;13(2):e0192667. doi: 10.1371/journal.pone.0192667 (PMC5813967; doi:10.1371/journal.pone.0192667)
Supplement: S1 File — (PDF) [file pone.0192667.s001.pdf]

Lipid surface concentration estimation, conversion of fluorescence intensity, and data analysis

In order to be able to fit the catalysis data with the kinetic model we proposed, the fluorescence intensity from YFP-PHGrp1 needs to be converted into the surface concentration of YFP-PHGrp1. For this purpose we used the total surface density of phosphatidylinositides in the model membrane as follows [1, 2]: For the PTEN catalysis experiments, bilayers were prepared which initially contained 0.2% PI(3,4,5)P3, which corresponds to 2850 PI(3,4,5)P3 molecules/μm^2 (or 4.75*10^{-6} μM m) in a monolayer. For this calculation, we assumed the average lipid cross section area to be ~0.7 nm^2, which corresponds to a total lipid surface concentration of 1.43*10^6 molecules/μm^2.

The fitting with the Langmuir adsorption model of measured YFP-PHGrp1 fluorescence intensities at different bulk concentrations (see Fig 2A) yielded the fluorescence intensity at surface saturation, $I^{\mathrm{R}}_{\mathrm{max}}$. Since at saturation each PI(3,4,5)P3 molecule has recruited one YFP-PHGrp1 molecule, $I^{\mathrm{R}}_{\mathrm{max}}$ corresponds to a surface concentration of 2850 YFP-PHGrp1 molecules/μm^2. To determine the surface density ρ of YFP-PHGrp1 in kinetics measurements in Figs 2B, 5, and 6A, the measured and background-corrected fluorescence intensities $I_{(\mathrm{t})}$ were multiplied with the conversion factor $\frac{2850 molecules}{\mu m^2} / I^{\mathrm{R}}_{\mathrm{max}}$ to obtain the surface density. In order to be able to also correct for daily variability of TIRF laser beam incident angles and intensity, we further scaled the surface density by a factor consisting of the ratio of two fluorescence intensities measured at the same bulk concentration (in this case of 300 nM). The initial fluorescence intensities I^{300}_{ini} (before PTEN or buffer injection) at a given bulk concentration (here 300 nM) is determined from averaging N measurements (typically $N \geqq 3$, but always $N \geqq 2$).

Accordingly, the time-dependent surface density in an experiment with 300 nM bulk concentration of YFP-PHGrp1, was determined as follows:

$$\rho_{300} = I_{(t)} * \frac{2850\,\frac{molecules}{\mu m^2}}{I_{\max}^{\mathrm{R}}} \frac{I_{eq}^{300,\mathrm{R}}}{I_{\mathrm{ini}}^{300}}$$

Two potential artifacts need to be considered. The first is the concern that PI(3,4,5)P3 may leach from the bilayer, thus changing the number of binding sites in the membrane. For example, Carvalho et al. indicated that PI(4,5)P2 leaches from phosphatidylcholine lipid bilayers over time [3]. Thus it is plausible that PI(3,4,5)P3 has the capacity to leach from the membrane as well. Secondly, YFP-PHGrp1 may nonspecifically bind to the membrane or to membrane defects. Both of these potential artifacts would compromise the determination of surface densities.

The supported lipid bilayers were used in this study within a few hours after preparation. To test whether PI(3,4,5)P3 leaches from the bilayer during this period, we compared YFP-PHGrp1 fluorescence intensities at the membrane right after preparation and 4 hours later (Figure A in S1 File). From the essentially identical fluorescence intensities, we conclude that negligible PI(3,4,5)P3 leaching occurred. The difference between this and Carvalho's result may be explained by the different phosphatidylinositide content in the bilayer, as their bilayers had an over ten times higher phosphatidylinositide content. An additional potentially contributing factor is the different sample preparation times. Carvalho et al., observed significant phosphatidylinositol loss after 24 hours, while we always completed our measurements during the course of 4 hours.

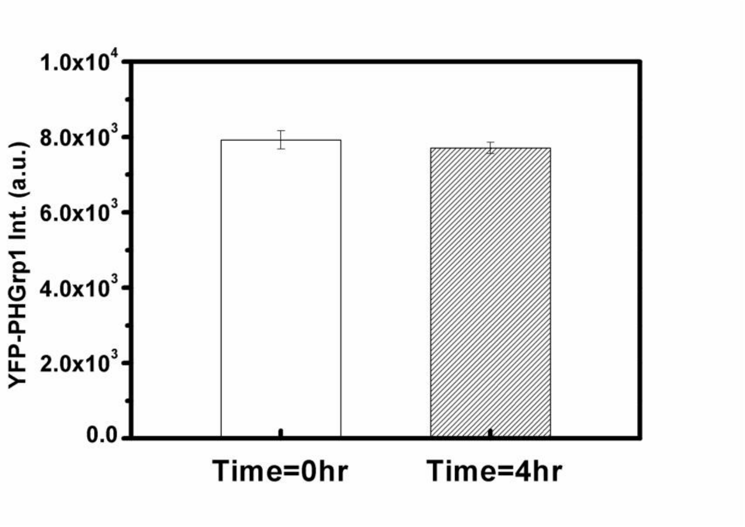

Fig A. YFP-PHGrp1 binding on the membrane after 0 and 4 hours of preparation, respectively. The membrane composition: 0.2% PI(3,4,5)P3 + 5% DOPS + 0.6% PI(4,5)P2 + 94% DOPC + 0.2% TR-DHPE. The bulk YFP-PHGrp1 concentration is 300 nM. Error bars represent the standard errors of the mean (SEM) for N = 3 measurements.

To address the question of non-specific binding, we equilibrated bilayers with YFP-PHGrp1 under near-saturating conditions (800 nM). We then hydrolysed PI(3,4,5)P3 via PTEN to remove all specific binding sites. Therefore, the remaining YFP-PHGrp1 signal would be caused by nonspecific binding. After background correction, we found that the percentage of YFP-PHGrp1 that remains on the bilayer after PI(3,4,5)P3 hydrolysis is only 2.53±0.15 % (SEM, three measurements on the same sample) of the initial binding (Figure B in S1 File), excluding significant contributions from non-specific binding to the determination of Grp1 surface densities.

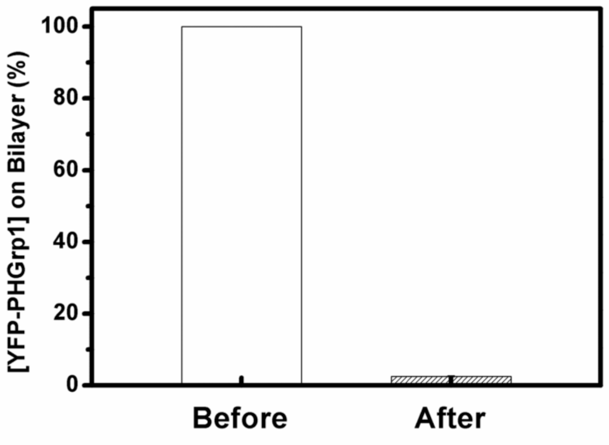

Fig B. YFP-PHGrp1 binding on the membrane before and after PTEN addition, respectively. The membrane composition: 0.2% PI(3,4,5)P3 + 5% DOPS + 0.6% PI(4,5)P2 + 94% DOPC + 0.2% TR-DHPE. The bulk YFP-PHGrp1 concentration is 800nM. The nonspecific binding of YFP-PHGrp1 on the model membrane is 2.53±0.15 %. Error bars represent the standard errors of the mean (SEM) for N = 3 measurements.

The global fitting of YFP-PHGrp1 association and dissociation kinetics to the compartment model was done via MATLAB (using the ode23s solver). Here, the fitting parameters were the association constant $k_{\mathrm{a}}^{\mathrm{Grp1}}$, dissociation constant $k_{\mathrm{d}}^{\mathrm{Grp1}}$, transport coefficient k_{tr}, and the height of the inner compartment h. These four parameters were fixed for the later PTEN model fitting. Based on the fitted value of $k_{\mathrm{a}}^{\mathrm{Grp1}}$ and $k_{\mathrm{d}}^{\mathrm{Grp1}}$, we determined the equilibrium dissociation constant K_{d} = 95.24±15.68 nM ($K_{\mathrm{d}} = \frac{k_{\mathrm{d}}^{\mathrm{Grp1}}}{k_{\mathrm{a}}^{\mathrm{Grp1}}}$, , the uncertainty of the K_{d} comes from the error propagation of standard error of $k_{\mathrm{d}}^{\mathrm{Grp1}}$and $k_{\mathrm{a}}^{\mathrm{Grp1}}$), which is comparable to the K_{d} value determined by equilibrium binding isotherm (Fig 2A, K_{d} = 126.51±6.07 nM). The

kinetic curves of YFP-PHGrp1 dissociation upon PTEN injection at different PI(4,5)P2 or YFP-PHGrp1 (Figs 5 and 6A) were first adjusted based on the K_{d} determined by the kinetic fitting, and then globally fitted with the kinetic model (Fig 4, Eqs. 1-7 in S3 File) again using MATLAB's ode23s solver. To assess how well each parameter is determined from fitting, we estimated the error by varying one parameter each time while fixing the rest of parameters at their optimum values and calculating the chi-square $\chi^2 = \sum_{i=1}^{N} \frac{1}{\sigma_i^2} (y_{fit} - y_{exp})^2$, where $\sigma_i^2 = \frac{1}{N} \sum_{i=1}^{N} (y_{fit} - y_{exp})_{opt}^2$ and N is the number of degrees of freedom. The chi-square χ^2 value is then plotted by varying each fitting parameter, and fit with a parabola to estimate the uncertainty of each fitting parameter when chi-square χ^2 increases by $\sqrt{2N}$ [4-6].

Alternatively to the model used to obtain the parameters shown in Table1, the kinetic curves of PTEN mediated YFP-PHGrp1 dissociation were fitted based either on a "recruitment only" model (Eqs. 1-5, 10, 11 in S3 File) or "allosteric activation only" model (Eqs. 1-4, 8, 9 in S3 File).

The following parameters were used as input for the solver:

$\mathrm{PTEN_{B\_sol}}$ (the initial PTEN bulk concentration); $\mathrm{Grp1_{B\_sol}}$ (the initial Grp1 bulk concentration); the initial PI(3,4,5)P3 concentration; the initial PI(4,5)P2 concentration.

The fitting parameters used in the analysis routine were:

In Fig 2B: $k_{\mathrm{a}}^{\mathrm{Grp1}}$, $k_{\mathrm{d}}^{\mathrm{Grp1}}$, k_{tr}, h

Figs 5 and 6A: $k_{\mathrm{cat}}^{\mathrm{PTEN}}$, $k_{\mathrm{a}}^{\mathrm{PTEN}}$, $k_{\mathrm{d}}^{\mathrm{PTEN}}$, $k_{\mathrm{a}}^{\mathrm{PTEN-PI(4,5)P_2}}$, $k_{\mathrm{d}}^{\mathrm{PTEN-PI(4,5)P_2}}$, $k_{\mathrm{cat}}^{\mathrm{PTEN-PI(4,5)P_2}}$, $k_{\mathrm{M}}^{\mathrm{PTEN}}$, $k_{\mathrm{M}}^{\mathrm{PTEN-PI(4,5)P_2}}$, $K_{\mathrm{PTEN,PI(4,5)P_2}}$, n

Fig 9A: $k_{\mathrm{cat}}^{\mathrm{PTEN}}$, $k_{\mathrm{a}}^{\mathrm{PTEN}}$, $k_{\mathrm{d}}^{\mathrm{PTEN}}$, $k_{\mathrm{a}}^{\mathrm{PTEN-PI(4,5)P_2}}$, $k_{\mathrm{d}}^{\mathrm{PTEN-PI(4,5)P_2}}$, $k_{\mathrm{M}}^{\mathrm{PTEN}}$

Fig 9B: $k_{\mathrm{cat}}^{\mathrm{PTEN}}$, $k_{\mathrm{a}}^{\mathrm{PTEN}}$, $k_{\mathrm{d}}^{\mathrm{PTEN}}$, $k_{\mathrm{cat}}^{\mathrm{PTEN-PI(4,5)P_2}}$, $k_{\mathrm{M}}^{\mathrm{PTEN}}$, $k_{\mathrm{M}}^{\mathrm{PTEN-PI(4,5)P_2}}$, $K_{\mathrm{PTEN,PI(4,5)P_2}}$, n

Resulting values for each fitting parameter are listed in Table 1.

References

1. He J, Haney RM, Vora M, Verkhusha VV, Stahelin RV, Kutateladze TG. Molecular mechanism of membrane targeting by the GRP1 PH domain. J Lipid Res. 2008;49(8):1807-15. doi: 10.1194/jlr.M800150-JLR200. PubMed PMID: WOS:000257566400022.
2. Ni T, Kalli AC, Naughton FB, Yates LA, Naneh O, Kozorog M, et al. Structure and lipid-binding properties of the kindlin-3 pleckstrin homology domain. Biochem J. 2017;474:539-56. doi: 10.1042/bcj20160791. PubMed PMID: WOS:000393770400006.
3. Carvalho K, Ramos L, Roy C, Picart C. Giant Unilamellar Vesicles Containing Phosphatidylinositol(4,5) bisphosphate: Characterization and Functionality. Biophys J. 2008;95(9):4348-60. doi: 10.1529/biophysj.107.126912. PubMed PMID: WOS:000260072600027.
4. Bevington PR, Robinson DK. Data reduction and error analysis for the physical sciences. Boston [u.a.]: McGraw-Hill; 2010.
5. Box GEP, Hunter WG, Hunter JS. Statistics for experimenters : an introduction to design. 1978.
6. Costa KD, Kleinstein SH, Hershberg U. Biomedical Model Fitting and Error Analysis. Sci Signal. 2011;4(192). doi: 10.1126/scisignal.2001983. PubMed PMID: WOS:000295257400004.
