# Supplementary material for: Kinetics of PTEN-mediated PI(3,4,5)P3 hydrolysis on solid supported membranes
Source: PLoS One. 2018 Feb 15;13(2):e0192667. doi: 10.1371/journal.pone.0192667 (PMC5813967; doi:10.1371/journal.pone.0192667)
Supplement: S2 File — (PDF) [file pone.0192667.s002.pdf]

Dead time measurement

To establish the speed with which the YFP-PHGrp1 reaches the desired bulk concentration through continuous flow in our measurement chamber, we prepared a supported lipid bilayer without PI(3,4,5)P3 (composition 5% DOPS + 94.8% DOPC + 0.2% TR-DHPE) on a glass cover slip to prevent non-specific binding of YFP-PHGrp1. Then we injected 600 nM YFP-PHGrp1 with different flow rates and observed the resulting fluorescence signal in the solution through confocal microscopy imaging. The data was fitted with a single exponential function and the inverse rate constant was defined as the dead time of the flow chamber. The flow rate for our experiments described in the main text was 1 cm/s, so the dead time was ~2 s.

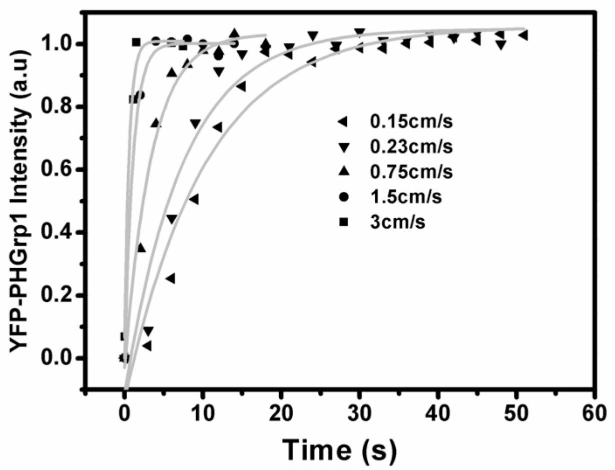

Fig A. Dead time of flow chamber measurement. 600 nM YFP-PHGrp1 was injected into chamber with a flow rate of 0.15 (left-pointing triangle), 0.23 (down-pointing triangle), 0.75 (triangle), 1.5 (circle), 3 (square) cm/s, respectively. A supported lipid bilayer (5% DOPS + 94.8% DOPC + 0.2% TR-DHPE) was spread on the glass surface to prevent non-specific binding of YFP-PHGrp1. The YFP-PHGrp1 signal in solution is monitored by confocal

microscopy. The kinetic data was fit with single exponential function: t_{mix} = 10.29±1.34 s (0.15 cm/s), 7.46±0.95 s (0.23 cm/s), 3.41±0.43 s (0.75 cm/s), 1.07±0.09 s (1.5 cm/s), and 0.58±0.14 s (3 cm/s).
