# Supplementary material for: Kinetics of PTEN-mediated PI(3,4,5)P3 hydrolysis on solid supported membranes
Source: PLoS One. 2018 Feb 15;13(2):e0192667. doi: 10.1371/journal.pone.0192667 (PMC5813967; doi:10.1371/journal.pone.0192667)
Supplement: S3 File — (PDF) [file pone.0192667.s003.pdf]

# Differential equations of the PTEN kinetic model

## Association & dissociation reactions

$$\frac{\partial[Grp1-PI(3,4,5)P3]_m}{\partial t}=k_a^{Grp1}[Grp1]_{comp\_sol}[PI(3,4,5)P3]_m-k_d^{Grp1}[Grp1-PI(3,4,5)P3]_m \qquad \textbf{[S1]}$$

$$\frac{\partial[Grp1]_{comp\_sol}}{\partial t}=\frac{1}{h}\{k_{tr}(Grp1_{B\_sol}-Grp1_{comp\_sol})+k_d^{Grp1}[Grp1-PI(3,4,5)P3]_m$$

$$-k_a^{Grp1}[Grp1]_{comp\_sol}[PI(3,4,5)P3]_m\} \qquad \textbf{[S2]}$$

$$\frac{\partial[PTEN]_m}{\partial t}=k_a^{PTEN}[PTEN]_{comp\_sol}-k_d^{PTEN}[PTEN]_m \qquad \textbf{[S3]}$$

$$\frac{\partial[PTEN]_{comp\_sol}}{\partial t}=\frac{1}{h}\{k_{tr}(PTEN_{B\_sol}-PTEN_{comp\_sol})-k_a^{PTEN}[PTEN]_{comp\_sol}+k_d^{PTEN}[PTEN]_m\} \qquad \textbf{[S4]}$$

$$\frac{\partial[PTEN-PI(4,5)P2]_m}{\partial t}=k_a^{PTEN-PI(4,5)P2}[PTEN]_{comp\_sol}[PI(4,5)P2]_m$$

$$-k_d^{PTEN-PI(4,5)P2}[PTEN-PI(4,5)P2]_m \qquad \textbf{[S5]}$$

## Enzyme dephosphorylation reactions

$$\frac{\partial[PI(3,4,5)P3]_m}{\partial t}=-k_{cat}^{PTEN}[PTEN]_m\{\frac{[PI(3,4,5)P3]_m}{K_M^{PTEN}+[PI(3,4,5)P3]_m}\}$$

$$-k_{cat}^{eff}[PTEN-PI(4,5)P2]_m\{\frac{[PI(3,4,5)P3]_m}{K_M^{PTEN-PI(4,5)P2}+[PI(3,4,5)P3]_m}\}$$

$$+k_d^{Grp1}[Grp1-PI(3,4,5)P3]_m-k_a^{Grp1}[Grp1]_{comp\_sol}[PI(3,4,5)P3]_m \qquad \textbf{[S6]}$$

$$\frac{\partial[PI(4,5)P2]_m}{\partial t}=k_{cat}^{PTEN}[PTEN]_m\{\frac{[PI(3,4,5)P3]_m}{K_M^{PTEN}+[PI(3,4,5)P3]_m}\}$$

$$+k_{cat}^{eff}[PTEN-PI(4,5)P2]_m\{\frac{[PI(3,4,5)P3]_m}{K_M^{PTEN-PI(4,5)P2}+[PI(3,4,5)P3]_m}\}$$

$$-k_a^{PTEN-PI(4,5)P2}[PTEN]_{comp\_sol}[PI(4,5)P2]_m+k_d^{PTEN-PI(4,5)P2}[PTEN-PI(4,5)P2]_m$$

**[S7]**

where $k_{cat}^{eff}=k_{cat}^{PTEN-PI(4,5)P2}\{\frac{[PI(4,5)P2]_m{}^n}{(K_{PTEN,PI(4,5)P2})^n+[PI(4,5)P2]_m{}^n}\}$

Meaning of subscripts:

a. m: protein or lipids are on the membrane

b. comp_sol: protein in the inner compartment of chamber

c. B_sol: protein in the bulk solution

## a. Allostery only model (S1-S4, S8, S9 Eqs.)

$$\frac{\partial[PI(3,4,5)P3]_m}{\partial t}=-k_{cat}^{PTEN}[PTEN]_m\{\frac{[PI(3,4,5)P3]_m}{K_M^{PTEN}+[PI(3,4,5)P3]_m}\}$$

$$-k_{cat}^{PTEN-PI(4,5)P2}(\frac{[PI(4,5)P2]_m{}^n}{(K_{PTEN,PI(4,5)P2})^n+[PI(4,5)P2]_m{}^n})(\frac{[PTEN]_m[PI(3,4,5)P3]_m}{K_M^{PTEN-PI(4,5)P2}+[PI(3,4,5)P3]_m})$$

$$+k_d^{Grp1}[Grp1-PI(3,4,5)P3]_m-k_a^{Grp1}[Grp1]_{comp\_sol}[PI(3,4,5)P3]_m$$

**[S8]**

$$\frac{\partial[PI(4,5)P2]_m}{\partial t}=k_{cat}^{PTEN}[PTEN]_m\{\frac{[PI(3,4,5)P3]_m}{K_M^{PTEN}+[PI(3,4,5)P3]_m}\}$$

$$+k_{cat}^{PTEN-PI(4,5)P2}\left(\frac{[PI(4,5)P2]_m{}^n}{(K_{PTEN,PI(4,5)P2})^n+[PI(4,5)P2]_m{}^n}\right)\left(\frac{[PTEN]_m[PI(3,4,5)P3]_m}{K_M^{PTEN-PI(4,5)P2}+[PI(3,4,5)P3]_m}\right\})$$

**[S9]**

## b. Recruitment only model (S1-S5, S10, S11 Eqs.)

$$\frac{\partial[PI(3,4,5)P3]_m}{\partial t}=-k_{cat}^{PTEN}\{[PTEN]_m+[PTEN-PI(4,5)P2]_m\}\{\frac{[PI(3,4,5)P3]_m}{K_M^{PTEN}+[PI(3,4,5)P3]_m}\}$$

$$+k_d^{Grp1}[Grp1-PI(3,4,5)P3]_m-k_a^{Grp1}[Grp1]_{comp\_sol}[PI(3,4,5)P3]_m$$

**[S10]**

$$\frac{\partial[PI(4,5)P2]_m}{\partial t}=k_{cat}^{PTEN}\{[PTEN]_m+[PTEN-PI(4,5)P2]_m\}\{\frac{[PI(3,4,5)P3]_m}{K_M^{PTEN}+[PI(3,4,5)P3]_m}\}$$

$$-k_a^{PTEN-PI(4,5)P2}[PTEN]_{comp\_sol}[PI(4,5)P2]_m+k_d^{PTEN-PI(4,5)P2}[PTEN-PI(4,5)P2]_m$$

**[S11]**
