# Supplementary material for: Kinetics of PTEN-mediated PI(3,4,5)P3 hydrolysis on solid supported membranes
Source: PLoS One. 2018 Feb 15;13(2):e0192667. doi: 10.1371/journal.pone.0192667 (PMC5813967; doi:10.1371/journal.pone.0192667)
Supplement: S4 File — (PDF) [file pone.0192667.s004.pdf]

Parameters in the PTEN kinetic model discussion

a. How to compare the association rate k_a of Grp1 to the literature value

Lai et al. used a stopped-flow technique with FRET readout to measure association and dissociation rate constants of PHGrp1 binding to PI(3,4,5)P3 [1]. However, they defined the association rather constant based on the accessible PIP concentration rather than Grp1 concentration. To compare their findings with our result, a conversion is required. According to the Langmuir adsorption model, ligand binding kinetics is described as:

$$[AB] = \frac{k_{on}[A]}{k_{on}[A]+k_{off}} B_{\max}(1-e^{-(k_{on}[A]+k_{off})t}) \qquad k_{obs} = k_{on}[A]+k_{off} \qquad \textbf{[S12]}$$

$[A]$: Solution ligand concentration

$B_{\max}$: Maximum binding site on the surface

$[AB]$: Surface concentration of ligand-receptor complex

The above formula is usually being used to describe ligand binding kinetics in SPR experiments, where the total surface receptor concentration is fixed while varying the solution ligand concentration. However, we can also fix the solution ligand concentration and vary the surface receptor concentration and to obtain the following formula:

$$[AB] = \frac{k_{on}[B]}{k_{on}[B]+k_{off}} A_{\max}(1-e^{-(k_{on}[B]+k_{off})t}) \qquad k_{obs} = k_{on}[B]+k_{off} \qquad \textbf{[S13]}$$

The observed rate constant for binding kinetics described by Eq. S12 and S13 are identical. Accordingly, we can convert it by multiplying k_{on} by the PI(3,4,5)P3 concentration and divide by the Grp1 concentration. For a lipid composition of PC / Dansyl-PE / PI(3,4,5)P3 = 92:5:3, k_{on} was found [1] to be 0.58 $\mu M^{-1}\ s^{-1}$ (with PIP3 concentration) which converts to 2.95 $\mu M^{-1}\ s^{-1}$ (with Grp1 concentration). For a second composition used in that study: PC / PS / Dansyl-PE /

PI(3,4,5)P3 = 69:23:5:3, k_{on} was found [1] to be 2.04 s^{-1} μM^{-1} (with PIP3 concentration) which converts to 11.94 μM^{-1} s^{-1} (with Grp1 concentration). Accordingly, the association rate constant of 1.68 μM^{-1} s^{-1} that we obtained here (see table 1) is more similar to the association rate constant determined in ref [1] in the absence of PS lipids, which is reasonable given the low PS content of our membranes (5%).

b. How to compare the association rate constant k_a of PTEN (unit: m/s) to the literature value?

We obtained an association rate constant of PTEN to the membrane of k_a^{PTEN} = 1.67*10^{-3} m/s. This quantity is an effective adsorption rate constant, which corresponds to the association constant times the lipid concentration. So the association constant can be expressed by the effective adsorption rate constant divided by the lipid surface concentration as follows:

$$k_{on}(s^{-1}\mu M^{-1}) = k_a^{PTEN}(m/s)/[PC+PS](\mu M \cdot m)$$

Our membrane contains 5% DOPS and ~94% DOPC, which corresponds to 1.18*10^{-4} µM m and 2.22*10^{-3} µM m, respectively. With the help of the above formula we obtained the association constant of k_{on} = 0.71 μM^{-1} s^{-1}, which is comparable to the literature value of 0.5 μM^{-1} s^{-1} [2].

c. Combination of Hill equation and Michaelis-Menten equation

It has been shown that PI(4,5)P2 can allosterically activate the catalytic activity of PTEN towards PI(3,4,5)P3. To quantitatively account for this allosteric effect, we divide the PI(3,4,5)P3 hydrolysis pathways into two pathways: membrane-bound PTEN unbound to PI(4,5)P2 on the one hand, and membrane-bound and PI(4,5)P2-bound PTEN (PTEN-PI(4,5)P2).

According to a Campbell et al. study [3], the dose-dependent activation of PTEN towards PI(3,4)P2 (or PI(3,4,5)P3) substrate in solution can be expressed as

$$V = \frac{V_{act}[PI(4,5)P2]}{K_{act} + [PI(4,5)P2]} \quad \textbf{[S14]}$$

where V is the initial rate of PI(3,4)P2 (or PI(3,4,5)P3) hydrolysis by PTEN. The rate V_{act} of PI(3,4)P2 hydrolysis by PTEN can also described by Michaelis-Menten kinetics as a function of PI(3,4)P2 (substrate). So V_{act} should be written as:

$$V_{act} = \frac{k_{act}[PTEN][PI(3,4)P2]}{K_M + [PI(3,4)P2]} \quad \textbf{[S15]}$$

Combining Eq. 14 and 15, one obtains

$$V = (\frac{k_{act}[PTEN][PI(3,4)P2]}{K_M + [PI(3,4)P2]})(\frac{[PI(4,5)P2]}{K_{act} + [PI(4,5)P2]}) \quad \textbf{[S16]}$$

Equivalently, in our theoretical model, the rate of PI(3,4,5)P3 hydrolysis by PTEN-PI(4,5)P2 is assumed to follow:

$$k_{cat}^{eff}[PTEN - PI(4,5)P2]_m\{\frac{[PI(3,4,5)P3]_m}{K_M^{PTEN-PI(4,5)P2} + [PI(3,4,5)P3]_m}\} \quad \textbf{[S17]}$$

where $$k_{cat}^{eff} = k_{cat}^{PTEN-PI(4,5)P2}\{\frac{[PI(4,5)P2]_m{}^n}{(K_{PTEN,PI(4,5)P2})^n + [PI(4,5)P2]_m{}^n}\} \quad \textbf{[S18]}$$

Eq. S15 and S17 have similar form except that we use the more general form of the Hill equation to describe the activation of PTEN by PI(4,5)P2 on the membrane [4].

In summary, the rate of PI(3,4,5)P3 hydrolysis by PTEN-PI(4,5)P2 in our model is assumed to follow Michaelis-Menten kinetics, but the turnover number k_{cat} is PI(4,5)P2-dependent.

References

1. Lai CL, Srivastava A, Pilling C, Chase AR, Falke JJ, Voth GA. Molecular Mechanism of Membrane Binding of the GRP1 PH Domain. Journal of Molecular Biology. 2013;425(17):3073-90. doi: DOI 10.1016/j.jmb.2013.05.026. PubMed PMID: WOS:000323860300005.

2. Das S, Dixon JE, Cho WW. Membrane-binding and activation mechanism of PTEN. Proc Natl Acad Sci U S A. 2003;100(13):7491-6. doi: DOI 10.1073/pnas.0932835100. PubMed PMID: WOS:000183845800018.

3. Campbell RB, Liu FH, Ross AH. Allosteric activation of PTEN phosphatase by phosphatidylinositol 4,5-bisphosphate. J Biol Chem. 2003;278(36):33617-20. doi: DOI 10.1074/jbc.C300296200. PubMed PMID: WOS:000185047500003.

4. Ullrich SJ, Hellmich UA, Ullrich S, Glaubitz C. Interfacial enzyme kinetics of a membrane bound kinase analyzed by real-time MAS-NMR. Nat Chem Biol. 2011;7(5):263-70. doi: 10.1038/nchembio.543. PubMed PMID: WOS:000289617800009.
